# Biodiversity of NSLAB in Traditional Serbian Cheeses and Stepwise Functional Characterization of Selected Autochthonous *Lactobacillus* Strains Through Technological, Probiotic and Intestinal Epithelial Interaction Assessment

**DOI:** 10.3390/foods15183220

**Published:** 2026-09-11

**Authors:** Nikola Bajčetić, Jovana Nenadović, Ivana Perić, Dušan Stevanović, Nemanja Mirković, Nikola Popović, Milica Mirković

**Affiliations:** 1 Department of Industrial Microbiology, Faculty of Agriculture, University of Belgrade, Nemanjina 6, 11080 Belgrade, Serbia; nikola.bajcetic@agrif.bg.ac.rs (N.B.); jovana.nenadovic@agrif.bg.ac.rs (J.N.); ivana.peric@agrif.bg.ac.rs (I.P.); nemanja.mirkovic@agrif.bg.ac.rs (N.M.); 2 Group for Probiotics and Microbiota-Host Interaction, Department of Microbiology and Plant Biology, Institute of Molecular Genetics and Genetic Engineering, University of Belgrade, Vojvode Stepe 444a, 11042 Belgrade, Serbia; dusan.stevanovic@imgge.bg.ac.rs (D.S.); nikola.popovic@imgge.bg.ac.rs (N.P.)

**Keywords:** autochthonous LAB, functional starter, probiotic characterization, autophagy

## Abstract

Traditional cheeses represent complex microbial ecosystems and valuable reservoirs of autochthonous lactic acid bacteria (LAB) with functional potential. This study characterized the biodiversity of non-starter LAB (NSLAB) isolated from traditional Serbian raw-milk household cheeses and selected promising autochthonous lactobacilli through a stepwise approach combining a safety assessment, technological characterization, probiotic evaluation and an in vitro interaction with Caco-2 intestinal epithelial cells. From 56 presumptive LAB isolates, RAPD-PCR differentiated 37 distinct genotypes, all of which were taxonomically identified by 16S rRNA gene sequencing. In line with the specific focus of this study on autochthonous lactobacilli, all seven isolates taxonomically assigned to this genus were selected for further characterization. Safety assessment showed that two strains (28.6%) formed biofilms and were excluded, whereas the remaining five isolates complied with EFSA antibiotic susceptibility criteria. These candidates exhibited strain-dependent degradation of αs1- and β-casein fractions and moderate milk acidification. Four strains survived simulated gastrointestinal transit, showed antagonistic activity against foodborne pathogens and adhered to Caco-2 cells without inducing cytotoxicity. *Lactiplantibacillus plantarum* ZI237 showed the broadest autophagy-related transcriptional response and *NQO1* induction. *Limosilactibacillus fermentum* KT upregulated *MUC2*, *MUC5*, *NQO1* and *HBD-1* but reduced Occludin protein abundance, indicating a mixed epithelial response. Both strains reduced p62 protein levels, supporting modulation of autophagy-related pathways. Overall, *Lpb. plantarum* ZI237 emerged as the most promising candidate, whereas *Lm. fermentum* KT requires further evaluation before application in functional dairy products.

## 1. Introduction

Traditional cheeses are dairy products produced in rural households and represent a long-standing tradition and an important part of cultural heritage [1]. Their production is usually based on raw cow, goat or sheep milk and is carried out without the addition of commercial starter cultures [2]. These dairy products are characterized by a richer, more intense flavor than cheeses made from pasteurized or microfiltered milk, which is partly attributed to microbial activity during ripening [3]. Microbiological diversity in traditional cheeses is shaped by multiple factors, including the origin and quality of milk, animal nutrition, geographical region, production method and hygiene conditions [4,5]. Numerous studies on artisanal dairy products from rural areas of the Western Balkans have shown that non-starter lactic acid bacteria (NSLAB), including genera such as *Lactobacillus*, *Lactococcus*, *Enterococcus*, *Streptococcus*, *Pediococcus*, *Leuconostoc* and *Weissella*, constitute the dominant component of the cheese microbiota [2,6,7,8,9]. Depending on their homo- or heterofermentative pathways, they convert fermentable carbohydrates into organic acids (mainly lactic acid), as well as carbon dioxide and ethanol, thereby significantly influencing acidification [10]. Through proteolytic and lipolytic processes, they promote the formation of peptides, free amino acids, fatty acids and aromatic compounds, which affect the sensory properties of cheeses [11]. Some NSLAB strains have also been shown to produce antimicrobial compounds such as hydrogen peroxide and bacteriocins, thus providing bioprotection of the product [12]. In recent years, increasing attention has been paid to the selection of lactic acid bacteria (LAB) that combine technological performance with probiotic-related properties, as such strains can contribute to the development of new functional products that, in addition to their nutritional value, may provide additional health benefits [13,14]. Candidates for such applications should be characterized within the framework of combined technological and probiotic characterization. From a technological perspective, desirable properties include acidification capacity, proteolytic activity and contribution to flavor and texture development [15]. Probiotic properties vary considerably among bacterial isolates and are commonly screened in vitro by evaluating their survival under simulated gastrointestinal conditions, adhesion to intestinal epithelial cells and antagonistic activity against pathogenic microorganisms. However, these functional properties must be considered together with safety-related characteristics, including accurate taxonomic identification, susceptibility to clinically relevant antibiotics and the absence of potentially harmful traits. Isolates fulfilling these preliminary functional and safety requirements may be regarded as potential probiotic candidates, whereas their qualification as probiotics requires confirmation of a health benefit in the host [16]. The genus *Lactobacillus* is among the most extensively studied and commercially applied probiotic groups, and many lactobacilli have a long history of safe use in fermented foods, supporting their selection as promising candidates [17]. Traditional cheeses represent particularly valuable reservoirs of lactobacilli with potential technological and probiotic relevance [3]. During cheese ripening, these microorganisms are exposed to multiple selective pressures, including low pH values, elevated NaCl concentrations, reduced water activity, limited availability of fermentable carbohydrates, and intense microbial competition [3,18]. Persistence under these conditions may reflect the stress tolerance of individual isolates and their capacity to activate adaptive response mechanisms. As adaptation to one environmental stress may confer cross-protection against other challenges, cheese-associated lactobacilli represent candidates for investigating tolerance to stresses encountered during food processing and gastrointestinal transit [18]. Studies indicate that probiotics can interact with intestinal epithelial cells and modulate host cell pathways involved in epithelial homeostasis [19]. Autophagy is an intracellular recycling mechanism that plays an important role in antimicrobial defense, regulation of intestinal immunity and maintenance of epithelial barrier integrity [20]. Selected strains can also affect mechanisms associated with the mucosal barrier, including the expression of mucin, which is necessary for the formation of the mucus layer and protection of the intestinal barrier, as well as innate epithelial defense through the modulation of antimicrobial peptides such as human β-defensin [21,22]. In addition, lactobacilli have been shown to modulate oxidative stress-related responses in intestinal epithelial models [23,24,25]. This study aimed to characterize the biodiversity of NSLAB isolated from traditional Serbian cheeses and to select promising autochthonous *Lactobacillus* strains by combining safety evaluations, technological characterizations, probiotic characterizations, and in vitro host–microbe interaction assessments using the Caco-2 intestinal epithelial cell model. Lactobacilli were chosen as the target group for subsequent functional screening because they represent one of the most extensively investigated groups of probiotic candidates, with broad application in functional dairy products. Such an approach broadens current knowledge of the microbial resources associated with traditional Serbian cheeses and expands the collection of autochthonous lactobacilli with potential application in functional products.

## 2. Materials and Methods

### 2.1. Sampling and Bacterial Isolation

Traditional cheeses made from raw cows or goats milk, produced by small-scale producers and rural households, were collected (purchased) from 11 different locations: Čačak (rural household Stojanović), Trstenik (small-scale producer SOLLO), Paraćin (rural household Žikić) (Central Serbia), Sremska Mitrovica (rural household Koziris), Vizić (small-scale producer Smoke and Cheese) (Northern Serbia), Valjevo (rural household Tadić), Lajkovac (rural household Nenadović), Zlatibor mountain region (rural household Todosijević) (Western Serbia), Kopaonik mountain region (small-scale producer Ukusi Kopaonika) (Southern Serbia), Vrbica (rural household Milojković) (Eastern Serbia) and Pirot (Dairy School Dr Obren Pejić) (Southeastern Serbia). After sampling, cheeses were transported to the Faculty of Agriculture of the University of Belgrade at 4 °C for further analysis.

The cheese samples were homogenized with sterile sodium citrate solution (2% *w*/*v*) in the Stomacher machine (Lab Blender Stomacher 400, Seward, Worthing, UK) for 3 min. Serial dilutions were prepared in sterile saline (0.85% NaCl), and the appropriate dilution was plated on the appropriate agar. *Lactobacillus* isolates were grown in De Man–Rogosa–Sharpe (MRS) medium (HiMedia Laboratories, Thane, India), at 37 and 42 °C under anaerobic conditions using the Gas-Pak Anaerobic System (Becton, Dickinson and Company, Franklin Lakes, NJ, USA). *Enterococcus*, *Lactococcus,* and *Streptococcus* isolates were grown in M17 medium (HiMedia Laboratories, Thane, India) with 0.5% (*w*/*v*) glucose (GM17) at 30 and 42 °C under aerobic conditions. *Leuconostoc* isolates were grown in MRS medium at 37 °C under aerobic conditions. Solid medium was prepared by adding agar (1.4%, *w*/*v*) (Torlak, Belgrade, Serbia) to broth medium. Randomly selected colonies were examined microscopically, and isolates were tested using Gram staining and catalase assays. Gram-positive and catalase-negative isolates were stored at −80 °C in appropriate broth containing 20% glycerol (*v*/*v*) for further analysis.

### 2.2. RAPD (Randomly Amplified Polymorphic DNA) Method

The isolation of DNA, the PCR method, and electrophoresis on agarose gel were performed according to the modified method [26]. After 24 h of incubation, one milliliter of culture grown in appropriate medium was centrifuged, and the cell pellet was washed twice with sterile Milli-Q water. Subsequently, 8–12 sterile glass beads (Glasperlen, 1.7–2.1 mm; Carl Roth, Karlsruhe, Germany) were added to the tube, and the suspension was shaken at 12,000 rpm for 30 min at 4 °C using an Eppendorf ThermoMixer C (Eppendorf SE, Hamburg, Germany). Following centrifugation, the resulting supernatant containing DNA was collected and used for RAPD analysis. The PCR products were generated using the RAPD primer (5′-CCGCAGCCAA-3′) [27]. Samples were amplified according to the following protocol: initial denaturation at 94 °C for 45 s, 30 °C for 2 min and 72 °C for 60 s, followed by 26 cycles at 94 °C for 5 s, 36 °C for 30 s, with an extension of 1 s for each cycle, 72 °C for 30 s, and then a final extension at 72 °C for 10 min. The PCR products were visualized with a 1.5% (*w*/*v*) agarose gel containing 1× TAE (Tris-acetate-EDTA) buffer using Midori Green Advance (Nippon Genetics Europe, Düren, Germany) at a constant voltage of 80 V. The gels were observed and photographed under a UV transilluminator (Biometra, Göttingen, Germany).

Cluster analysis of RAPD-PCR banding patterns was performed using PAST software (version 5.3) based on a binary presence/absence (1/0) matrix, and the dendrogram was generated using the Jaccard similarity coefficient and UPGMA clustering method.

### 2.3. Identification of the Selected Isolates

DNA isolation of the selected isolates was performed using the ZymoBIOMICS DNA Miniprep Isolation Kit (Zymo Research, Irvine, CA, USA) according to the manufacturer’s instructions.

The PCR program and primers for the amplification of the variable region of the *16S rRNA* gene, P1 16S (5′-GAGAGTTTGATCCTGGC-3′) and P2 16S (5′-AGGAGGTGATCCAGCCG-3′), were used [28]. All PCR products were purified using a QIAquick PCR Purification Kit (QIAGEN, Hilden, Germany). The isolates were identified at the sequencing center of Macrogen Sequencing Service, Netherlands. Sequence analysis was performed with the BLAST+ program using the NCBI database http://www.ncbi.nlm.nih.gov/BLAST (accessed on 15 April 2026).

### 2.4. Safety Assessment

#### 2.4.1. Biofilm Formation

The ability of selected strains to form biofilm was assessed with modifications [29]. The 24 h bacterial cultures grown in MRS broth at 37 °C and with 5% CO_2_ were adjusted to 0.5 McFarland turbidity in Hi-Sensitivity Test Broth (HiMedia, Thane, India). Aliquots of 200 μL were inoculated into sterile 96-well polystyrene microtiter plates and incubated at 37 °C with 5% CO_2_ for 24 h. Wells were gently washed three times with PBS, air-dried in an inverted position and stained with 1% crystal violet for 15 min. Excess stain was removed by washing, and bound dye was solubilized in an ethanol:acetone solution (80:20, *v*/*v*). Biofilm biomass was quantified at 595 nm (OD_595_) using the Tecan Infinite 200 Pro plate reader (MTX Lab Systems, Vienna, Austria). Based on the corrected OD values (sample OD_595_ − negative control OD_595_), the strains were classified into two groups: strains without biofilm-forming ability (OD_595_ < 0.2), and biofilm formers (OD_595_ ≥ 0.2) [30].

#### 2.4.2. Antibiotic Susceptibility

The minimal inhibitory concentration (MIC) of selected strains was determined using microdilution testing according to European Food Safety Authority (EFSA) guidance on the characterization of microorganisms used as feed additives [31]. The selected strains were tested against different antibiotics purchased from Sigma-Aldrich (St. Louis, MO, USA) in the following concentration ranges (µg/mL): ampicillin (1–8), gentamicin (8–64), kanamycin (32–128), streptomycin (32–128), erythromycin (0.5–2), clindamycin (2–8), tetracycline (2–64) and chloramphenicol (2–16). Antibiotics at appropriate concentrations were dissolved in Hi-Sensitivity Test Broth, and 180 μL of each antibiotic solution was dispensed into sterile 96-well polystyrene microtiter plates. Then, 20 μL of overnight cultures of selected lactobacilli, adjusted to 0.5 McFarland units in Hi-Sensitivity Test Broth, was added to each well. The microtiter plates were incubated at 37 °C with 5% CO_2_ for 24 h. After incubation, values were measured at 600 nm (OD_600_) using a Tecan Infinite 200 Pro plate reader (MTX Lab Systems, Vienna, Austria). The MIC was defined as the lowest antibiotic concentration at which no visible growth of the bacterial strain was observed.

### 2.5. Technological Characterization

#### 2.5.1. Proteolytic Activity

Proteolytic activity was assessed by an SDS–PAGE casein hydrolysis assay with minor modifications [32]. Fresh bacterial cells (10 mg with an approximate density of 1 × 10^10^ cells/mL) were resuspended in 20 mM sodium phosphate buffer (pH 6.8; NaH_2_PO_4_/Na_2_HPO_4_), and mixed in a 1:1 ratio with 5 mg/mL of αs1- and β-casein, respectively (Sigma, St. Louis, MO, USA), prepared in the same buffer. The mixtures were incubated for 3 h at 30 °C. The degradation of αs1- and β-casein was analyzed on 12.5% sodium dodecyl sulfate-polyacrylamide gel electrophoresis (SDS-PAGE).

#### 2.5.2. Acidification Capacity

The acidification capacity of the strains was monitored by adding 1 mL of 24 h bacterial cultures grown in MRS medium (containing 1 × 10^8^ CFU) to 100 mL of 10% *w*/*v* pasteurized, reconstituted skimmed milk (Merck, Darmstadt, Germany) at a temperature of 37 °C. The pH of the milk was measured after 2, 4, 6, 8 and 24 h using a pH meter (Mettler-Toledo International Inc., Greifensee, Switzerland).

### 2.6. Probiotic Characterization

#### 2.6.1. Survival Under the Simulated GIT Conditions

Survival of the tested isolates after simulated GIT transit was evaluated with modifications [33]. The 24 h grown cultures of selected strains were centrifuged (5000× *g*, 15 min) in a Centrifuge 5804R (Eppendorf, Hamburg, Germany), washed twice with PBS and concentrated ten times in reconstituted 10% (*w*/*v*) sterile skimmed milk. The bacterial suspensions were diluted 10 times with gastric juice (GJ)—125 mM NaCl, 7 mM KCl, 45 mM NaHCO_3_ and 0.3% pepsin (Sigma-Aldrich, St. Louis, MO, USA)—adjusted to pH 2.0 with 1 M HCl and incubated for 90 min at 37 °C with agitation (200 rpm) in a shaker (Marshall Scientific, Hampton, NH, USA). The suspensions were centrifuged (2050× *g*, 15 min), resuspended in duodenal juice (DJ)—1% bovine bile (Sigma-Aldrich, St. Louis, MO, USA)—adjusted to pH 8.0 with 0.1 M NaOH and incubated at 37 °C for 10 min. After centrifugation (2050× *g*, 15 min), the suspensions were resuspended in intestinal juice (IJ)—0.3% bovine bile (Sigma-Aldrich, St. Louis, MO, USA) and 0.1% pancreatin (Sigma-Aldrich, St. Louis, MO, USA)—adjusted to pH 8.0 with 0.1 M NaOH and incubated for 120 min at 37 °C with 5% CO_2_.

The number of surviving isolates was determined before treatment and after each treatment with GJ, DJ and IJ. Using the dilution method, the appropriate suspension was plated on MRS agar and incubated for 48 h at 37 °C with 5% CO_2_.

The percentage of surviving colonies was calculated using the formula:

% survival = (log CFUt/log CFUt0) × 100

where CFUt represents the number of viable cells after each step of treatment, and CFUt0 is the initial number of cells before exposure.

#### 2.6.2. Antimicrobial Characterization of Selected Strains

Indicator pathogenic strains (*Salmonella enterica* subsp. *enterica* serovar Typhimurium ATCC 14028, *Streptococcus pyogenes* ATCC 19615, *Staphylococcus aureus* ATCC 25923 and *Escherichia coli* ATCC 25922) were grown in LB (Luria–Bertani) medium (10 g/L tryptone, 5 g/L yeast extract and 5 g/L NaCl) and GM17 medium (*Listeria monocytogenes* ATCC19111 and *Listeria monocytogenes* IMS4 from the laboratory collection). Strains were incubated at 37 °C for 24 h with agitation (180 rpm) using a LabShaker (Adolf Kühner AG, Birsfelden, Switzerland), except for the *Listeria* sp. strains, which were grown under the same temperature conditions without shaking.

The 24 h cultures of the indicator pathogens, containing approximately 1 × 10^8^ CFU/mL, were incorporated into the appropriate top agar medium (LA or GM17 for *Listeria* spp.), which was subsequently spread over the surface of the corresponding agar plates. Wells of 6 mm diameter were made in the agar, and 50 µL of each *Lactobacillus* broth culture was added. Petri dishes were incubated for 24 h at 37 °C, and the presence of inhibition zones around the wells indicated the antimicrobial activity of the selected strains. In order to examine the production of antimicrobial compounds of proteinaceous nature, crystals of pronase E (Sigma Chemie GmbH, Deisenhofen, Germany) were placed close to the edge of the wells. TOP agar was prepared by adding agar (0.7%, *w*/*v*) (Torlak, Belgrade, Serbia) to broth medium.

### 2.7. Interaction with Human Intestinal Cells

#### 2.7.1. Cultivation of Caco-2 Cells

The human intestinal epithelial cell line Caco-2 (ECACC 86010202), obtained from the European Collection of Authenticated Cell Cultures, was cultured in DMEM (Gibco, Thermo Fisher Scientific, Waltham, MA, USA) containing 10% fetal bovine serum, 1% penicillin–streptomycin and 2 mM L-glutamine. The cells were grown at 37 °C in 5% CO_2_. At approximately 90% confluence, the monolayers were detached by incubation with a 1% Trypsin solution (Sigma-Aldrich, St. Louis, MO, USA) in PBS for 5 min at 37 °C. Then, 5  ×  10^5^ cells per well were seeded in 24-well plates and grown for 14 days. The medium was replaced every two days. The last medium change was performed without the addition of antibiotics.

#### 2.7.2. Adhesion Assay

The overnight cultures of selected lactobacilli, with a concentration of approximately 1 × 10^8^ CFU/mL (MOI100), were added to differentiated Caco-2 cells. The plates were incubated at 37 °C in 5% CO_2_ for 2 h. After incubation, Caco-2 cells were washed two twice with PBS, and 1% trypsin solution (Sigma Aldrich) was added to each well. The suspension was incubated for 5 min at 37 °C in 5% CO_2_, after which 100 µL of the cell suspension was collected and serially diluted, and aliquots were plated on MRS agar plates. The plates were incubated for 48 h at 37 °C in 5% CO_2_. The results are presented as the mean number of adherent cells to Caco-2, expressed in CFU/mL. From the remaining cells, total RNA and proteins were isolated and stored at −80 °C for real-time PCR analysis, and at −20 °C for Western blot analysis.

#### 2.7.3. Lactate Dehydrogenase (LDH) Cytotoxicity Assay

Cytotoxicity of the treatments was assessed by quantifying lactate dehydrogenase (LDH) activity in the supernatants of Caco-2 cells. LDH activity was measured spectrophotometrically using a plate reader Tecan Infinite 200 Pro with the CyQUANT LDH Cytotoxicity Assay Kit (Thermo Fisher Scientific, Waltham, MA, USA), as per the manufacturer’s instructions.

#### 2.7.4. RNA Isolation and qPCR

Total RNA extraction from Caco-2 cells was performed as previously described with modifications [34].

Total RNA was isolated using acid phenol (pH 4) extraction followed by isopropanol precipitation. The concentration and purity of the extracted RNA were determined using a BioSpec-nano spectrophotometer (Shimadzu Corporation, Kyoto, Japan). Reverse transcription was performed using 200 ng of total RNA according to the manufacturer’s instructions (Thermo Fisher Scientific, Waltham, MA, USA). Random hexamers (Applied Biosystems, Foster City, CA, USA) and RiboLock RNase inhibitor (Thermo Fisher Scientific, Waltham, MA, USA) were included in the reaction mixture. Quantitative PCR (qPCR) was performed under the following conditions: 2 min at 95 °C activation, 40 cycles of 5 s at 95 °C and 30 s at 60 °C in a Line-Gene 9600 Plus Real-Time PCR (Hangzhou Bioer Technology Co., Ltd., Hangzhou, China) by using the IC Green qPCR Universal Kit (NIPPON Genetics, Düren, Germany).

The primers used in this study are listed in Table 1. Gene expression levels were normalized to the endogenous control glyceraldehyde-3-phosphate dehydrogenase (*GAPDH*) gene and calculated using the 2^−ΔΔCt^ method.

#### 2.7.5. Western Blotting

Total proteins were extracted from Caco-2 cells using Radio-Immunoprecipitation Assay (RIPA) buffer (50 mM Tris-HCl, pH 7.4; 150 mM NaCl; 1% NP-40; 0.25% sodium deoxycholate) supplemented with protease inhibitor cocktail tablets (Roche, Basel, Switzerland) and 1 mM phenylmethylsulphonyl fluoride (Sigma-Aldrich, St. Louis, MO, USA), for 30 min on ice. The lysates were then centrifuged, and the supernatants were collected and stored at −20 °C until further analysis.

Protein concentration was determined using a BCA protein assay kit (Thermo Fisher Scientific, Waltham, MA, USA). Equal amounts of protein (10 µg) were separated by 12.5% sodium dodecyl sulfate polyacrylamide gel electrophoresis (SDS-PAGE). Electrophoresed proteins were transferred from the gel to a 0.2 mm nitrocellulose membrane (GE HealthCare, Chicago, IL, USA) using a Bio-Rad Mini *trans*-blot system (Bio-Rad, Hercules, CA, USA). Membranes were blocked with 5% non-fat dry milk in TBS-Tween (50 mM Tris-HCl, pH 7.4; 150 mM NaCl; and 0.05% Tween-20) for 1 h at room temperature followed by incubation with primary antibodies overnight at 4 °C. Primary antibodies against SQSTM1/p62, LC3 (MAP1LC3B), Beclin-1 (BECN1), Claudin-4 (CLDN4), Occludin (OCLN), E-cadherin (CDH1) and β-actin, used as a loading control, were obtained from Cell Signaling Technology (Danvers, MA, USA). After washing with TBS-Tween, the membranes were incubated with the appropriate horseradish peroxidase (HRP)-conjugated secondary antibody (Thermo Fisher Scientific, Waltham, MA, USA) for 1 h at room temperature. Chemiluminiscence was detected using ChemiDoc Touch Imaging System with Image Lab Touch Software (version 2.4; Bio-Rad, Hercules, CA, USA). The image resolution/sensitivity scale was set to 4  ×  4 pixel binning settings, and pictures were captured by using the Rapid Auto Exposure mode. The intensity of the bands was quantified in ImageJ (National Institutes of Health, NIH, Bethesda, MD, USA) software (version 1.54g). The results were normalized to β-actin used as a loading control, and values for non-treated Caco-2 cells were adjusted to 1 in order to calculate the fold change.

### 2.8. Statistical Analysis

Experiments were performed in triplicate, and the results are presented as means ± standard deviations (SDs). Statistical analysis was performed using GraphPad Prism software (version 8.4.3; GraphPad Software, San Diego, CA, USA). Differences in survival under simulated gastrointestinal conditions were analyzed by two-way analysis of variance (ANOVA) followed by Tukey’s multiple comparisons test, with comparisons performed between simulated GIT treatments for each strain. qPCR, adhesion, LDH cytotoxicity and Western blot densitometric data were analyzed using Student’s *t*-test. Differences were considered statistically significant at *p* < 0.05. Statistical significance was indicated as follows: * *p* < 0.05, ** *p* < 0.01, *** *p* < 0.001 and **** *p* < 0.0001.

## 3. Results

### 3.1. Cheese Sampling and Bacterial Isolation

Of the isolates obtained from 11 different traditional cheeses, 56 showed characteristic features of lactic acid bacteria (LAB) and were therefore selected for further analysis. All tested cultures were Gram-positive and appeared morphologically as single cells, chains or grouped cells. None of the 56 isolates showed catalase activity.

### 3.2. Analysis of RAPD-PCR Profiles

**Figure 1:**
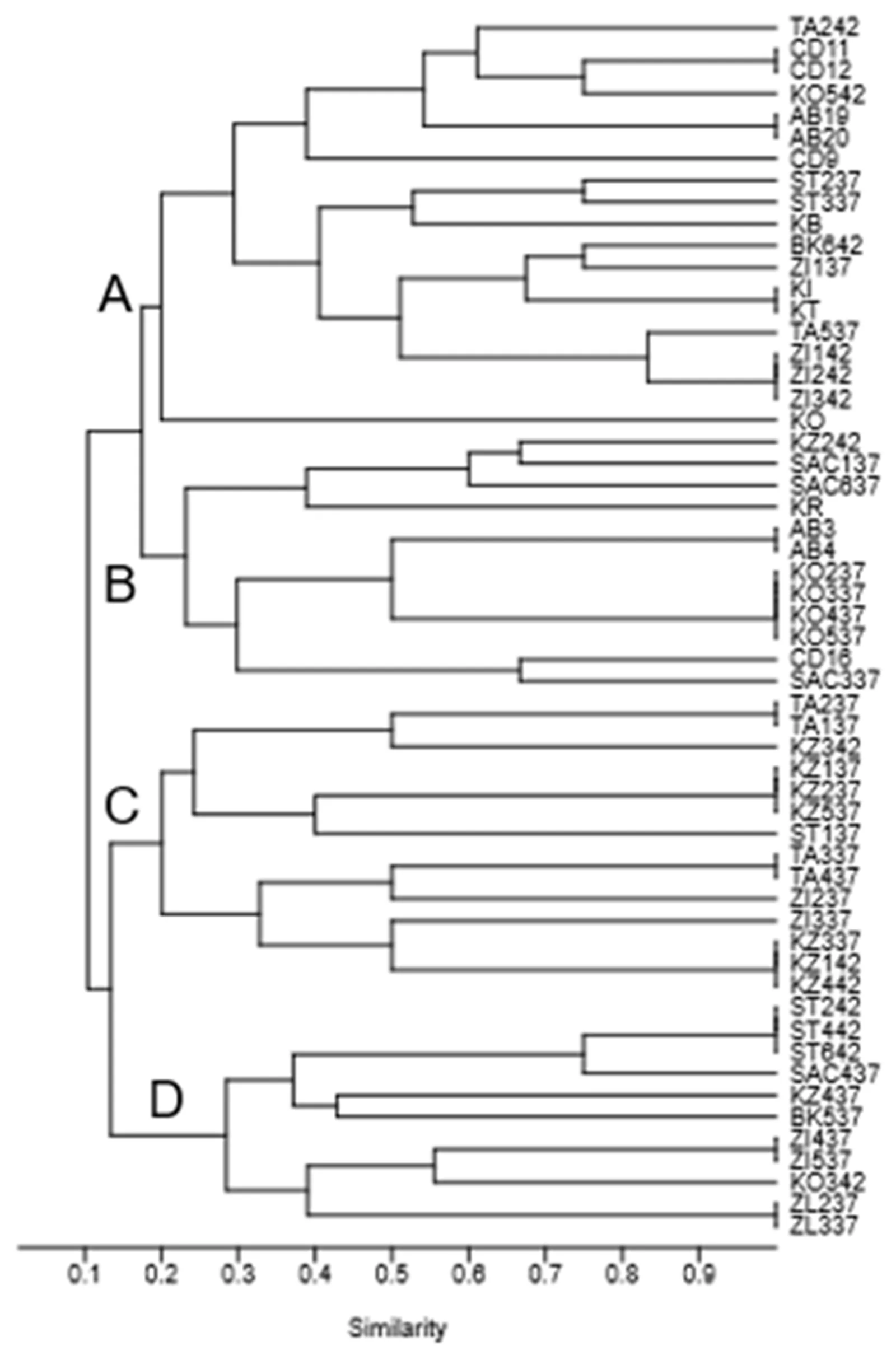
Dendrogram obtained from RAPD-PCR patterns of the analyzed isolates. The RAPD profiles were grouped into clusters according to the unweighted pair group method with arithmetic mean (UPGMA) analysis, based on Jaccard’s similarity coefficient. Isolates sharing a similarity coefficient of 1.0 were considered to have identical DNA fingerprints.

The RAPD-PCR profiles grouped the isolates into four major clusters, designated A–D, based on UPGMA analysis using Jaccard’s similarity coefficient (Figure 1). No clustering was observed according to milk type or geographical origin, as isolates from different sources were distributed in the same RAPD clusters, indicating considerable diversity among the obtained fingerprinting profiles.

Several isolates exhibited RAPD-PCR profiles that were indistinguishable under the applied experimental conditions, with a similarity coefficient of 1.0., including CD11/CD12, AB19/AB20, KI/KT and ZI142/ZI242/ZI342 in group A; AB3/AB4 and KO237/KO337/KO437/KO537 in group B; TA137/TA237, KZ137/KZ237/KZ537, TA337/TA437 and KZ337/KZ142/KZ442 in group C; and ST242/ST442/ST642, ZI437/ZI537 and ZL237/ZL337 in group D. To dereplicate the isolate collection, 19 isolates (33.93%) with RAPD-PCR profiles indistinguishable under the applied conditions were excluded from subsequent taxonomic analysis, and 37 representative isolates were subjected to 16S rRNA gene sequencing.

### 3.3. Identification of the Selected Isolates

The list of identified strains is presented in Table 2. According to the sequences of *16S rRNA* gene sequencing, 19 isolates belonged to *Enterococcus* spp. (51.35%): ten *Enterococcus faecium*, four *Enterococcus faecalis*, three *Enterococcus durans*, one *Enterococcus mundtii* and one *E. lactis*; five *Lactococcus* spp. (13.51%): four *Lactococcus lactis* and one *Lactococcus lactis* ssp. *cremoris*; five *Leuconostoc mesenteroides* sp. (13.51%); one *Streptococcus* sp. (2.70%); *Streptococcus macedonicus* and seven *Lactobacillus* spp. (18.92%) strains: three *Lactiplantibacillus plantarum*, two *Lacticaseibacillus paracasei*, one *Lacticaseibacillus rhamnosus* and one *Limosilactibacillus fermentum*, which were selected for further experiments. Accordingly, all seven strains assigned to lactobacilli were retained for subsequent functional characterization, with no further selection applied within this group, in line with the study focus on autochthonous lactobacilli.

### 3.4. Safety Assessment

**Figure 2:**
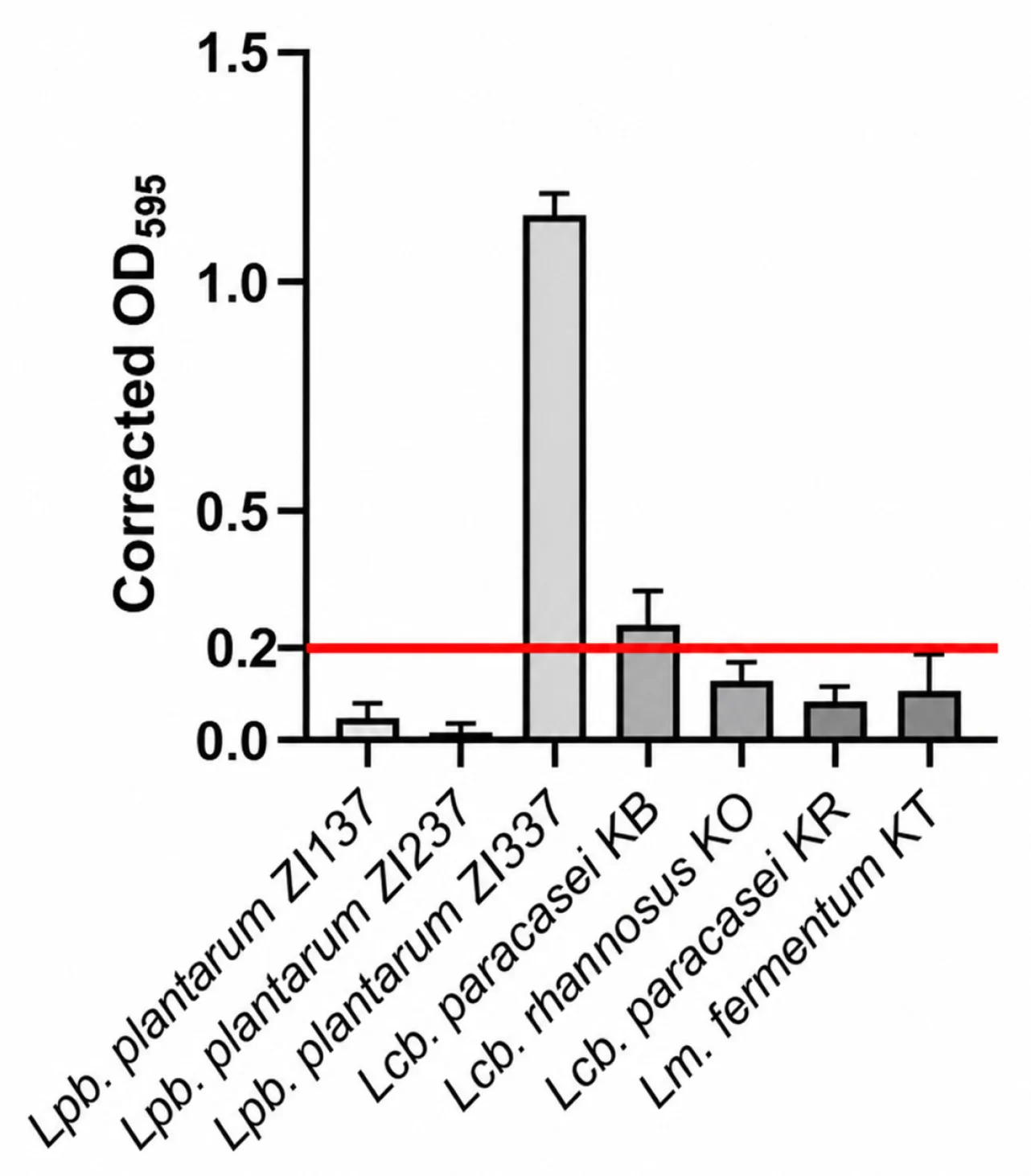
Biofilm formation ability of the tested *Lactobacillus* strains. Values were expressed as corrected OD_595_ values (sample OD595 − negative control OD_595_). The red horizontal line indicates the cut-off value for biofilm formation (OD_595_ = 0.2). Strains with values below 0.2 were classified as strains without biofilm-forming ability (OD_595_ < 0.2), and with values ≥ 0.2 as biofilm formers. Error bars indicate standard deviations (*n* = 6).

Based on the OD values measured at 595 nm and using 0.2 as the cut-off value, *Lpb. plantarum* ZI337 (1.14 ± 0.04) and *Lcb. paracasei* KB (0.25 ± 0.07) were identified as biofilm-forming strains and were excluded from further analysis, while the remaining strains were classified as non-biofilm formers (OD_595_ < 0.2) (Figure 2).

All five tested *Lactobacillus* strains were susceptible to the selected antibiotics. The MIC values are presented in Table 3. Considering their safety for use in food applications, the strains were subsequently evaluated for technological properties as potential starter cultures.

### 3.5. Technological Characterization

#### 3.5.1. Proteolytic Activity

**Figure 3:**
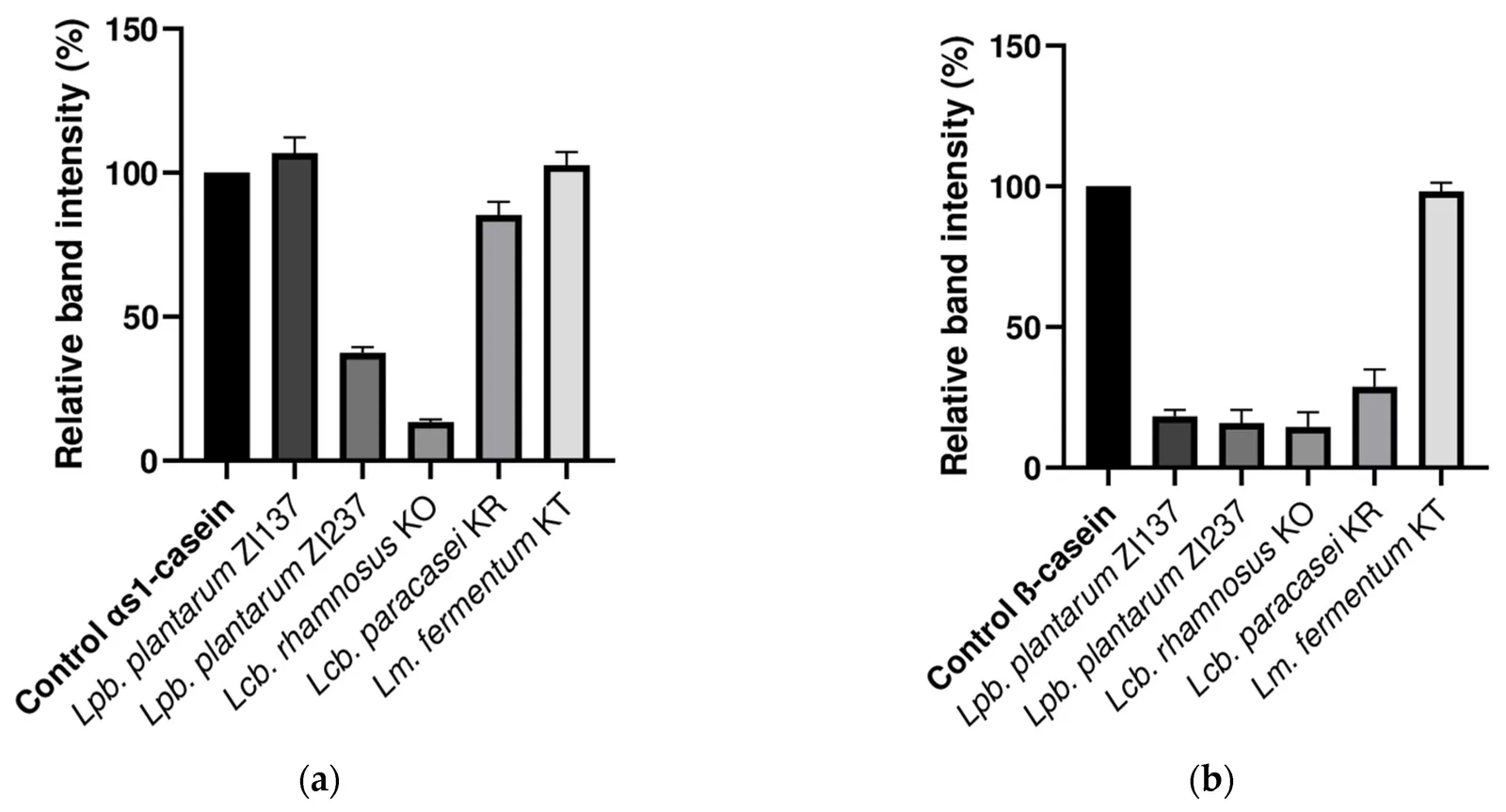
Proteolytic activity of selected lactobacilli toward αs1-casein and β-casein. Residual αs1-casein (**a**) and β-casein (**b**) band intensities were expressed relative to the untreated casein control, which was set to 100%. Error bars indicate SDs (*n* = 3).

The strains *Lpb. plantarum* ZI237 and *Lcb. rhamnosus* KO demonstrated activity towards αs1-casein. *Lcb. paracasei* KR exhibited minimal visible degradation, while *Lpb. plantarum* ZI137 and *Lm. fermentum* KT exhibited no detectable activity. *Lpb. plantarum* ZI237 showed a proteolytic effect towards β-casein, followed by *Lpb. plantarum* ZI137 and *Lcb. rhamnosus* KO. A visible but less pronounced reduction of the β-casein band was observed for *Lcb. paracasei* KR, while strain *Lm. fermentum* KT exhibited no detectable degradation of β-casein. These findings are presented in Figure 3.

#### 3.5.2. Acidification Capacity

**Figure 4:**
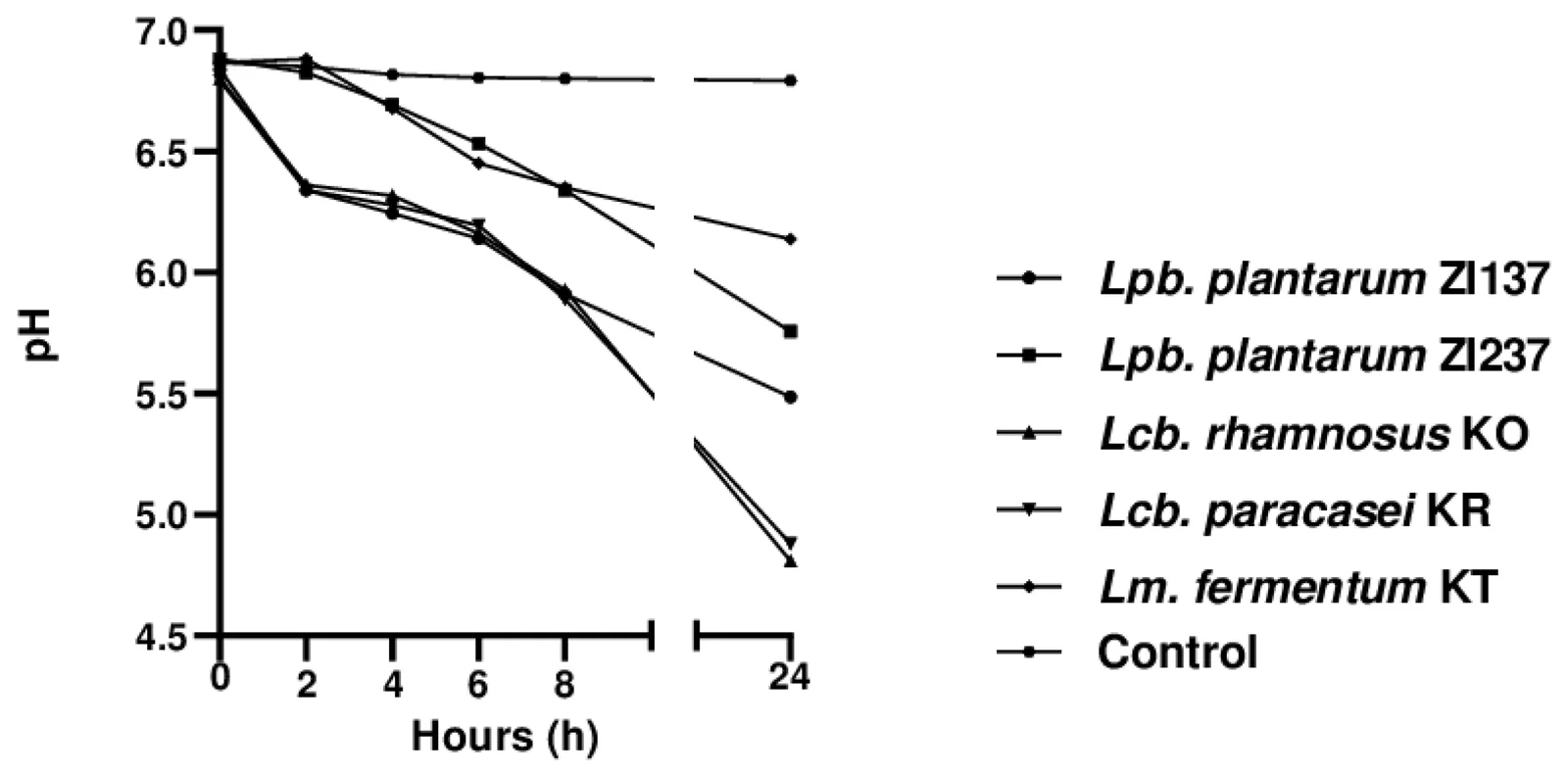
Acidification activity of selected *Lactobacillus* strains. Error bars are not clearly visible because the standard deviations were smaller than the symbols (*n* = 3).

The acidification capacity of the selected strains is shown in Figure 4. None of the tested strains showed the strong acidification activity expected from primary starter cultures either after 6 h or after 24 h, indicating that the strains have moderate acidification capacity. After 6 h of incubation, the decrease in pH ranged from 0.35 for *Lpb. plantarum* ZI237 (from 6.88 ± 0.01 to 6.53 ± 0.01) to 0.70 for *Lpb. plantarum* ZI137 (from 6.84 ± 0.01 to 6.14 ± 0.01). After 24 h of incubation, the decrease in milk pH ranged from 0.73 for *Lm. fermentum* KT (6.14 ± 0.01) to 1.99 for *Lcb. rhamnosus* KO (4.81 ± 0.01).

### 3.6. Probiotic Characterization

#### 3.6.1. Survival Under the Simulated GIT Conditions

Based on survival under simulated GIT conditions, four out of five tested strains showed the ability to survive through all gut sections, including gastric, duodenal and intestinal juice. The strain *Lacticaseibacillus paracasei* KR did not grow in any of the simulated conditions and was discarded from further testing.

**Figure 5:**
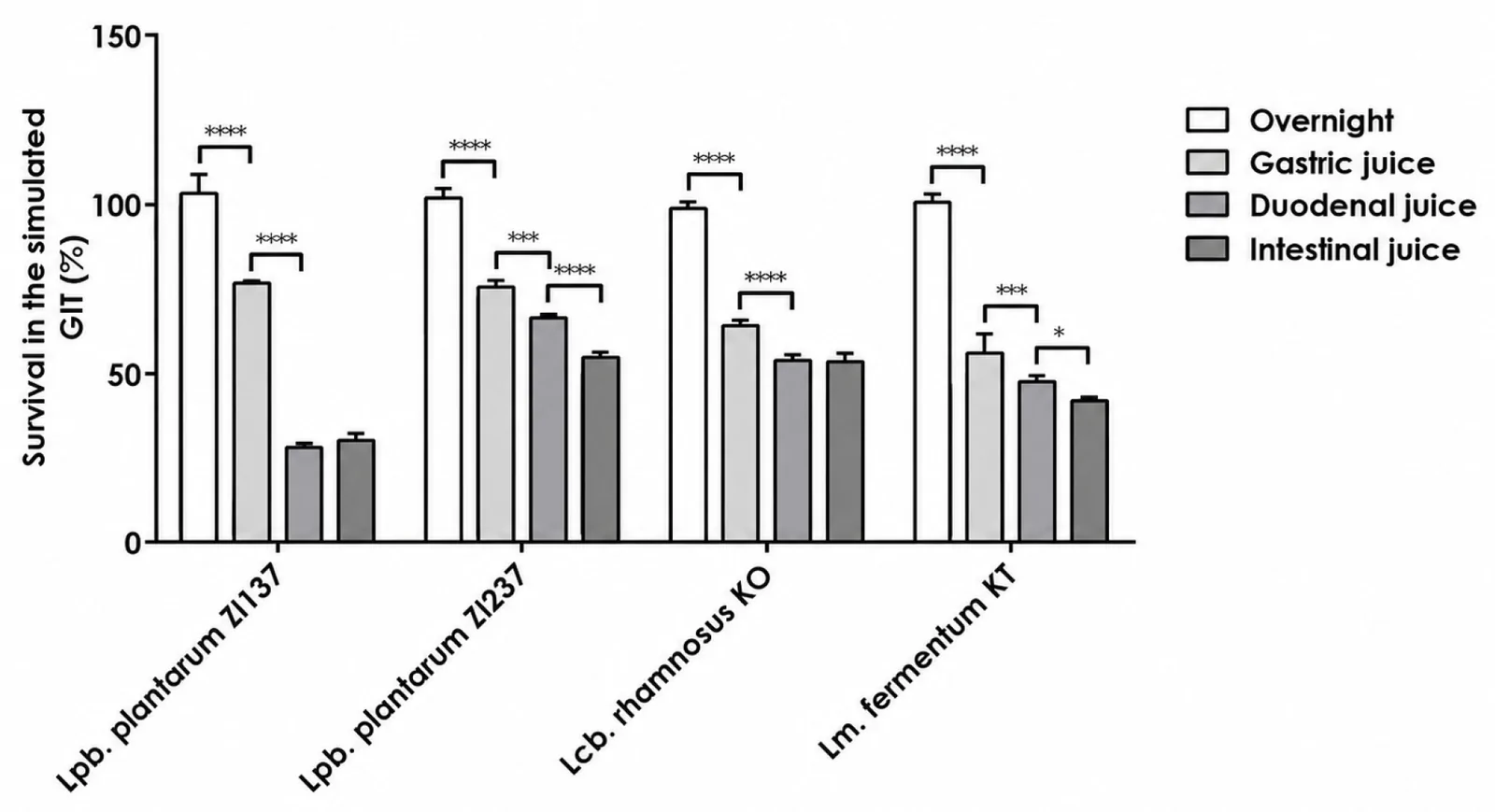
Survival percentage of four chosen *Lactobacillus* strains after transition through simulated gastrointestinal conditions: gastric juice, duodenal juice and intestinal juice. Statistical differences between simulated conditions are marked as a: *, ***, ****. Error bars indicate SDs (*n* = 3). Statistical comparisons were performed between different GIT treatments for each strain. Statistical significance: * *p* < 0.05, *** *p* < 0.001, **** *p* < 0.0001.

After treatment with gastric juice, all four strains showed survival percentages over 50%: *Lpb. plantarum* ZI137 76.63% ± 0.57, *Lpb. plantarum* ZI237 74.90% ± 1.18, *Lcb. rhamnosus* KO 66.10% ± 0.75 and *Lm. fermentum* 54.74% ± 1.05. After duodenal juice, the number of viable cells was reduced the most in *Lpb. plantarum* ZI137, which showed 27.44% survival, while *Lpb. plantarum* ZI237 maintained the highest value with 63.42% ± 0.16. *Lcb. rhamnosus* KO and *Lm. fermentum* showed similar survival rates with 52.84 ± 0.14 and 51.12 ± 0.14, respectively. After intestinal juice, *Lpb. plantarum* ZI137 remained the strain with the lowest survival rate (29.12% ± 0.17); *Lpb. plantarum* ZI237, with 55.92% ± 5.43, remained the most resistant strain; *Lcb. rhamnosus* KO and *Lm. fermentum* KT survived in similar percentages, with 46.53% ± 0.24 and 41.83% ± 0.75. The results are shown in Figure 5.

#### 3.6.2. Antimicrobial Activity

The inhibition zones of the four tested strains remained unaffected in the presence of pronase E, suggesting that antimicrobial activity was not associated with bacteriocin production. Among the tested strains, *Lpb. plantarum* ZI137, *Lpb. plantarum* ZI237 and *Lcb. rhamnosus* KO suppressed the growth of five out of six examined pathogens, whereas *Lm. fermentum* KT inhibited two out of six. The results are shown in Table 4.

### 3.7. Interaction with Human Intestinal Cells

#### 3.7.1. Adhesion and Cytotoxicity Assays

**Figure 6:**
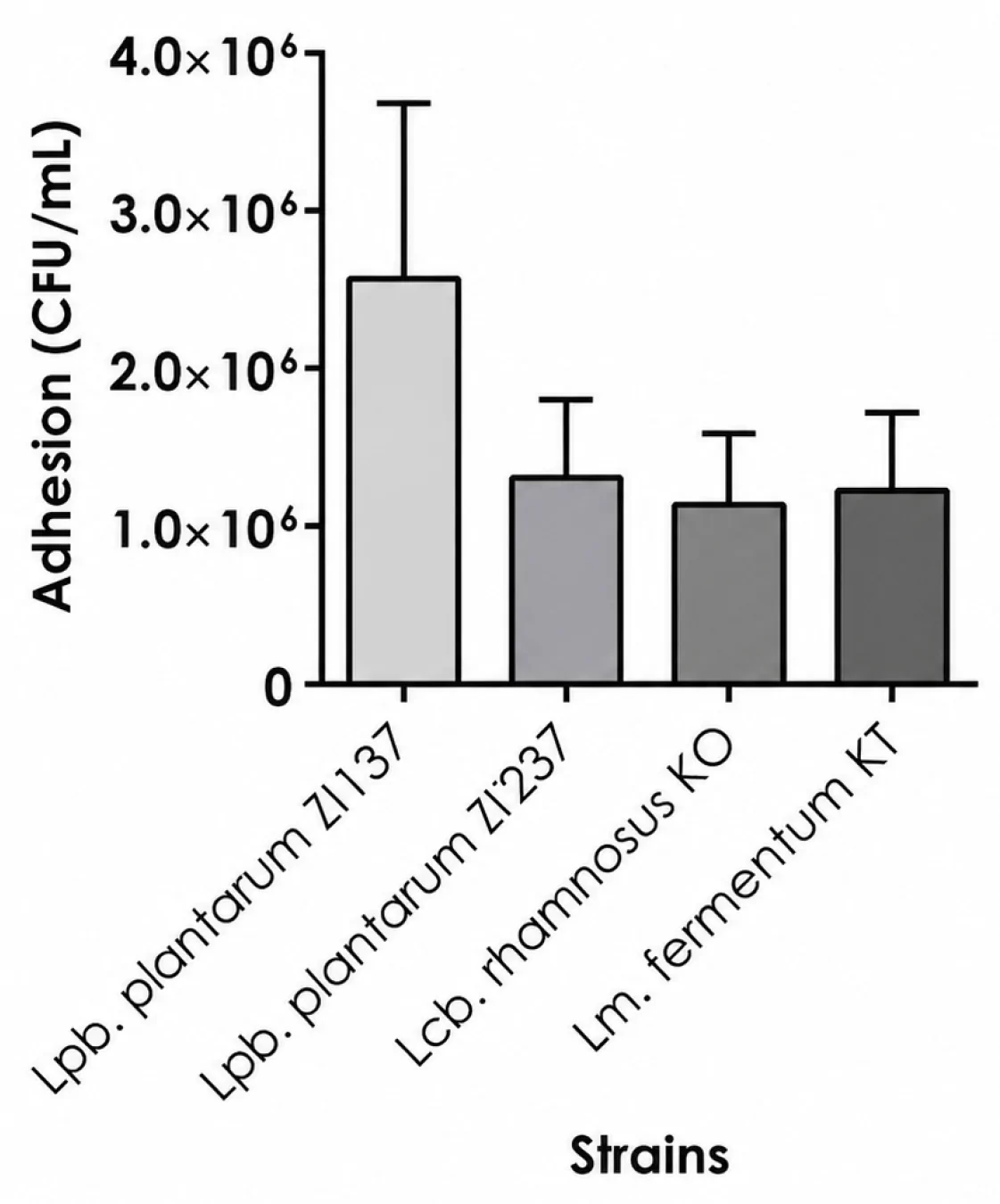
Adhesion of chosen *Lactobacillus* strains to Caco-2 cells (CFU/mL). Error bars indicate standard deviations (*n* = 3).

The adhesion capacity of the tested strains to Caco-2 cells is shown in Figure 6.

*Lpb. plantarum* ZI137 exhibited the highest adhesion, with approximately 2.6 × 10^6^ CFU/mL attached cells. *Lpb. plantarum* ZI237, *Lcb. rhamnosus* KO and *Lm. fermentum* KT showed adhesion levels ranging between 1.1 × 10^6^ and 1.3 × 10^6^ CFU/mL.

None of the tested strains showed cytotoxicity on Caco-2 cells compared with the positive control, as determined by the LDH assay. 

#### 3.7.2. Effect of Lactobacilli on the Expression of Autophagy-Related Genes and Proteins

**Figure 7:**
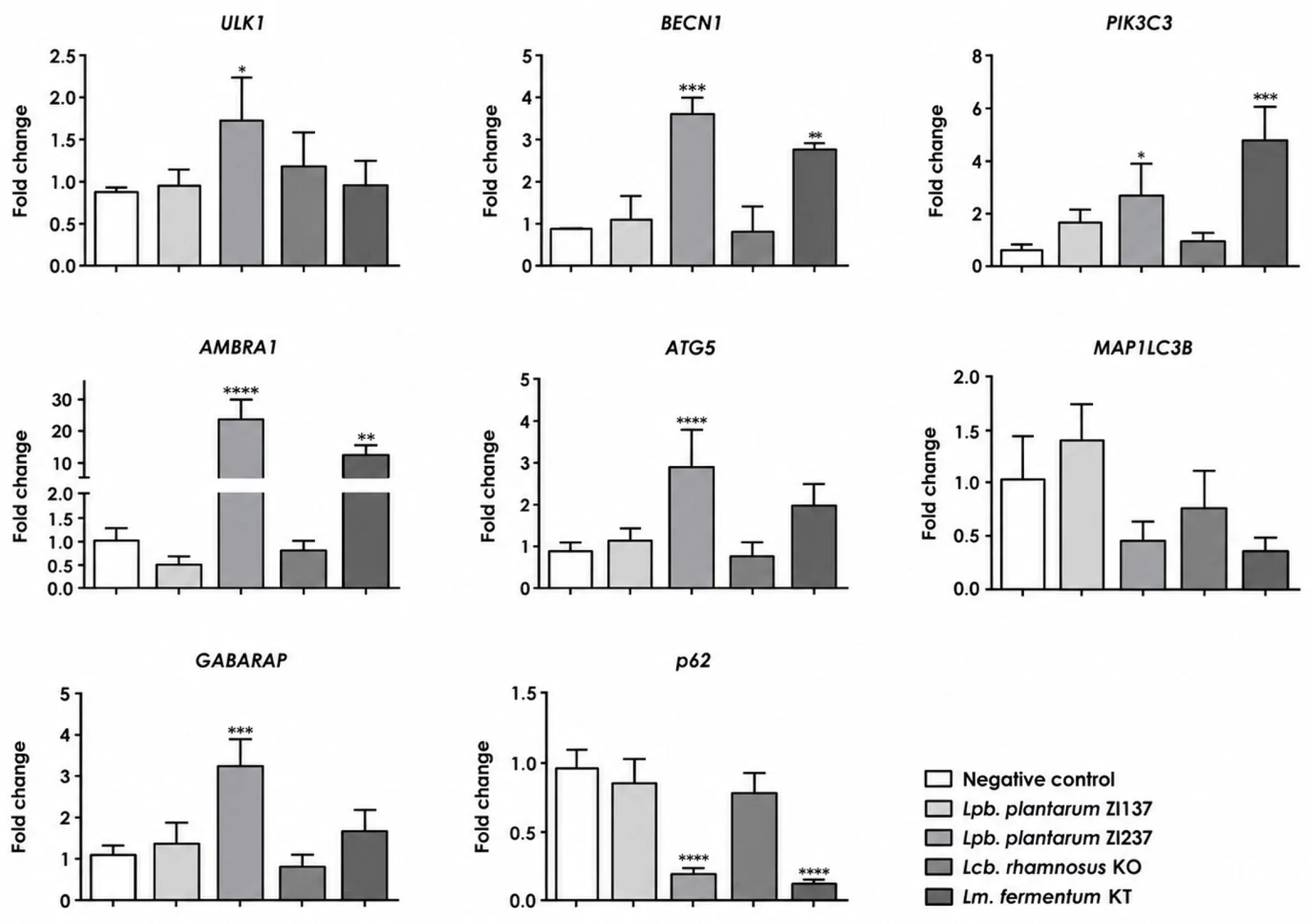
Expression levels of autophagy-related genes (*ULK1*, *BECN1*, *PIK3C3*, *AMBRA1*, *ATG5*, *MAP1LC3B*, *GABARAP* and *p62*) in Caco-2 cells following treatment with *Lactiplantibacillus plantarum* ZI137, *Lactiplantibacillus plantarum* ZI237, *Lacticaseibacillus rhamnosus* KO and *Limosilactobacillus fermentum* KT. Data are presented as the fold change relative to the negative control. Bars represent means ± SDs (*n* = 3). Statistical significance compared to the negative control: * *p* < 0.05, ** *p* < 0.01, *** *p* < 0.001, **** *p* < 0.0001.

The four tested strains gave different transcriptional responses to autophagy-related genes (Figure 7). The analyzed genes covered different stages of the autophagy pathway, including autophagy initiation (*ULK1*), phagophore formation (*BECN1*, *PIK3C3* and *AMBRA1*), autophagosome elongation and formation (*ATG5*, *MAP1LC3B* and *GABARAP*) and selective regulation of autophagy (*p62*). These results indicate that *Lpb. plantarum* ZI237 modulated the transcription of multiple genes involved in different stages of the autophagy pathway in epithelial cells. This strain significantly increased the expression of the *ULK1* gene, as well as the *BECN1*, *PIK3C3*, *AMBRA1, ATG5* and *GABARAP* genes, while *SQSTM1/p62* expression was significantly decreased. A similar, although less extensive pattern, was observed in *Lm. fermentum* KT. This strain significantly increased the expression of *BECN1*, *PIK3C3* and *AMBRA1*, and also strongly decreased the expression of *p62*. However, unlike *Lpb. plantarum* ZI237, *Lm. fermentum* KT did not significantly affect *ULK1*, *ATG5* or *GABARAP*. The remaining strains did not significantly affect the expression of the mentioned genes.

**Figure 8:**
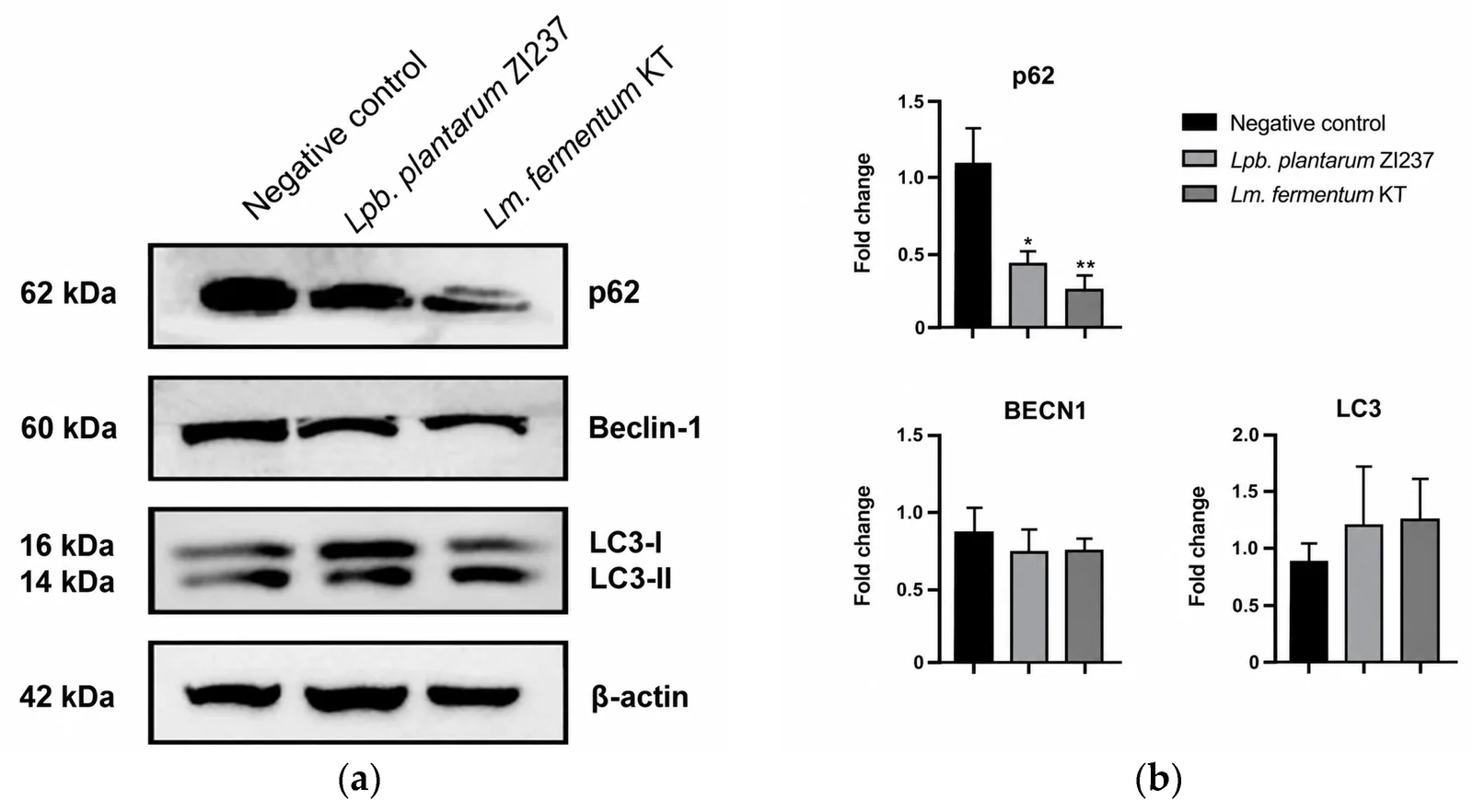
Western blot analysis of autophagy-related proteins in Caco-2 cells treated with selected lactobacilli. (**a**) Representative Western blot bands showing the protein expression of p62, Beclin-1 and LC3 in Caco-2 cells treated with *Lpb. plantarum* ZI237 and *Lm. fermentum* KT, compared with the negative control. β-actin was used as the loading control. Molecular weights are indicated on the left. (**b**) Densitometric analyses of p62, BECN1 and LC3 protein expression normalized to β-actin and presented as the fold change relative to the negative control. Data are shown as means ± SDs of three independent experiments. Statistical significance was determined relative to the negative control: * *p* < 0.05, ** *p* < 0.01.

Based on the qPCR findings, *Lpb. plantarum* ZI237 and *Lm. fermentum* KT were selected for further protein-level analysis by Western blotting (Figure 8). Treatment with both strains resulted in a significant reduction in p62 protein abundance, consistent with the decreased *p62* expression observed at the mRNA level. Full-length Western blot images are provided in Supplementary Materials.

#### 3.7.3. Effect of Lactobacilli on the Expression of Mucin-, Antimicrobial-, and Antioxidant-Related Genes

**Figure 9:**
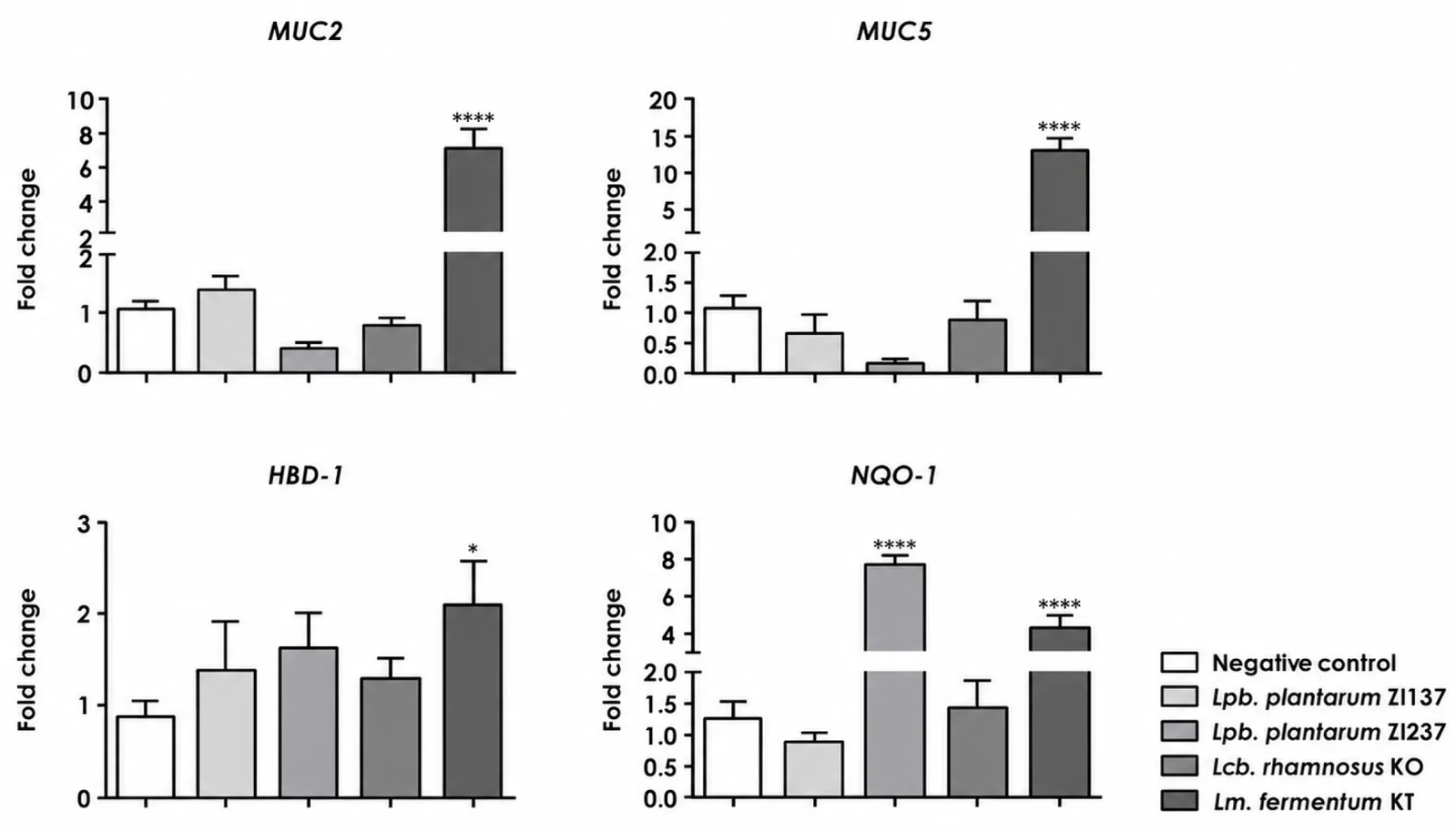
Expression levels of mucin genes (*MUC2* and *MUC5*), the antimicrobial peptide gene *HBD-1* and the antioxidant response gene *NQO1* in Caco-2 cells following treatment with *Lactiplantibacillus plantarum* ZI137, *Lactiplantibacillus plantarum* ZI237, *Lacticaseibacillus rhamnosus* KO and *Limosilactobacillus fermentum* KT. Data are presented as the fold change relative to the negative control. Bars represent means ± SDs (*n* = 3). Statistical significance compared to the negative control: * *p* < 0.05, **** *p* < 0.0001.

In the subsequent analysis, a panel of genes was selected to assess different functional aspects of the epithelial response, including mucin-mediated barrier function through mucin-related genes (*MUC2* and *MUC5*), antimicrobial epithelial defense through *HBD-1*, and antioxidant/cytoprotective response through *NQO1* (Figure 9). *Lm. fermentum* KT significantly upregulated *MUC2*, *MUC5*, *HBD-1* and *NQO1*, while *Lpb. plantarum* ZI237 significantly induced only *NQO1* expression. The remaining strains did not significantly affect the expression of the mentioned genes.

#### 3.7.4. Western Blot Analysis of Epithelial Barrier-Related Proteins

**Figure 10:**
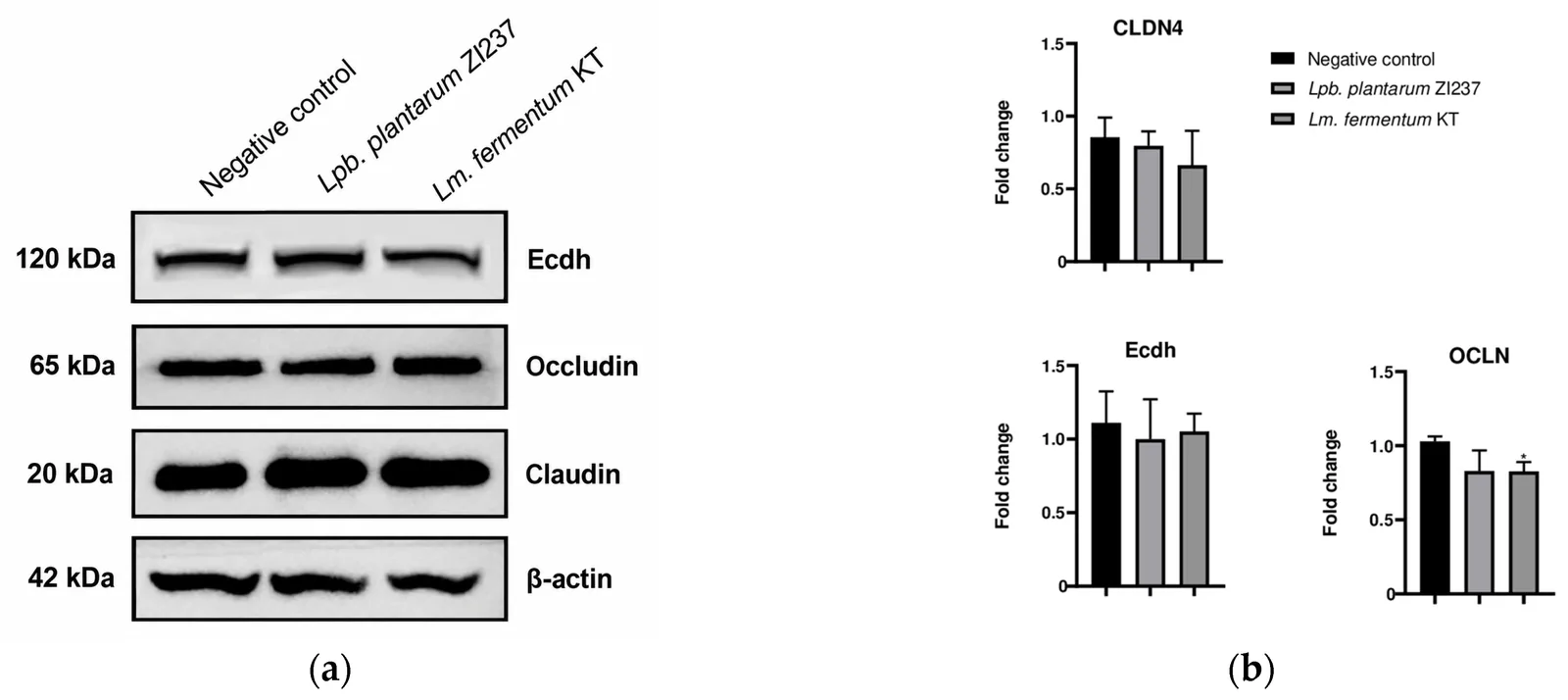
Western blot analysis of epithelial barrier-related proteins in Caco-2 cells treated with selected lactobacilli. (**a**) Representative Western blot bands showing the protein expression of E-cadherin (Ecdh), Occludin (OCLN) and Claudin-4 (CLDN4) in Caco-2 cells treated with *Lpb. plantarum* ZI237 and *Lm. fermentum* KT, compared with the negative control. β-actin was used as the loading control. Molecular weights are indicated on the left. (**b**) Densitometric analysis of protein expression normalized to β-actin and presented as the fold change relative to the negative control. Data are shown as the mean ± SD of three independent experiments. Statistical significance was determined relative to the negative control: * *p* < 0.05.

Based on the results obtained, *Lpb. plantarum* ZI237 and *Lm. fermentum* KT were selected for further Western blot analyses to assess the effects of these strains on the expression of E-cadherin, Claudin-4 and Occludin proteins, key proteins associated with epithelial barrier junctions responsible for maintaining epithelial barrier integrity (Figure 10). No significant changes in E-cadherin or Claudin-4 protein abundance were observed following treatment with either strain. In contrast, *Lm. fermentum* KT significantly reduced Occludin protein abundance compared with the negative control (*p* < 0.05). Full-length Western blot images are provided in the Supplementary Materials.

## 4. Discussion

The current findings support the view that traditional cheeses represent an important reservoir of diverse autochthonous lactic acid bacteria, which is in agreement with other published works [41,42], while the review by Terzić-Vidojević et al. [2] further highlighted the wide diversity of NSLAB in artisanal dairy products from the Western Balkan countries.

Although 13 isolates shared indistinguishable RAPD-PCR profiles at a similarity coefficient of 1.0 and were therefore assigned to the same genotype, the remaining isolates displayed distinct RAPD-PCR patterns, resulting in 37 genotypes among the 56 presumptive LAB isolates. The clustering of isolates from different geographical locations and milk types indicated no clear association between RAPD-PCR profiles and either source. Thus, the observed heterogeneity refers to the diversity of RAPD-PCR profiles across the entire isolate collection. These results are in line with previous reports describing RAPD-PCR as a simple method for assessing biodiversity in complex dairy ecosystems [43]. However, the methodological limitations of the RAPD-PCR method should be taken into account when interpreting the results. Its discriminatory power depends on the number and sequence of primers used, while its reproducibility can be affected by several factors such as DNA quality and concentration, reaction mixture composition, amplification parameters, thermal cycler performance and electrophoretic conditions [44]. Since only one primer was used in this study, isolates sharing a similarity coefficient of 1.0 should be considered indistinguishable under the experimental conditions used.

Although 16S rRNA gene sequencing has allowed taxonomic assignment of representative isolates, the conserved nature of this marker may limit its ability to discriminate between closely related species and strains. This limitation is particularly relevant for members of the former *Lactobacillus casei* group, *including Lacticaseibacillus paracasei* and *Lacticaseibacillus rhamnosus*, which may exhibit very similar 16S rRNA gene sequences [45]. The results showed that the identified isolates predominantly belonged to the genus *Enterococcus* (51.35%), with the dominant species being *E. faecium* (10 strains). Previously, the high number of enterococci reported in traditional products was attributed to poor hygiene and fecal contamination on farms [46], but now their widespread presence is attributed to proven resistance to various environmental selective pressures: high temperatures during milk pasteurization, low pH during ripening and high NaCl concentrations in cheese [47]. The presence of *E. faecium* as the dominant species in cheeses is consistent with the results of other studies [48,49,50]. *Lactococcus* spp. are mainly associated with the early stages of fermentation, while during ripening their numbers may decrease due to lactose reduction, NaCl addition and low pH [51]. A similar explanation can be applied to *Leuconostoc* spp., which are often associated with fresh and young traditional cheeses and may vary depending on the type of cheese and the ripening stage [2,9]. The only streptococcal isolate identified in this study was *Streptococcus macedonicus*, a species originally described from the traditional Greek cheese Kasseri and later recognized as a species often associated with artisanal fermented foods, especially those of dairy origin [52,53]. An interesting finding of this study was that lactobacilli were found in only two of the eleven cheeses analyzed, one goat cheese and one cow cheese, indicating their relatively limited distribution among the samples. *Lactiplantibacillus plantarum* was the dominant species (three strains), reported as one of the most abundant members of the isolated lactobacilli community [54,55,56]. Although strains belonging to other identified LAB genera may also exhibit probiotic-related properties, the subsequent functional characterization was focused on lactobacilli because they represent one of the most extensively investigated bacterial groups for probiotic applications, particularly in functional dairy products.

The ability of lactobacilli to form biofilms may have different roles, depending on the environment. In the gastrointestinal tract, biofilm-associated growth may promote bacterial persistence, competitive exclusion of pathogens, and localized production of antimicrobial metabolites [57]. Similarly, biofilms formed by well-characterized beneficial microorganisms may contribute to microbial stability and desirable biochemical transformations in some traditional fermented foods. However, uncontrolled biofilm formation in dairy processing environments poses an important technological and hygienic issue. Established biofilms may consequently act as reservoirs for recontamination of food products, potentially harboring spoilage microorganisms or foodborne pathogens and contributing to product spoilage, reduced shelf life, and contamination of food-processing equipment [58]. Therefore, although biofilm formation is not inherently indicative of pathogenicity, it was considered an undesirable trait within the conservative preliminary screening applied in this study. Given our intention to apply the selected strains in functional foods, two strains (*Lpb. plantarum* ZI337 and *Lcb. paracasei* KB) were excluded from further analysis due to their biofilm-forming properties. Although lactobacilli generally have GRAS status, there is growing concern about foodborne antibiotic resistance and its possible transmission through mechanisms such as horizontal gene transfer and the potential transfer of this resistance to other bacteria [59]. In this study, none of the strains showed MIC values exceeding the EFSA microbiological breakpoints for the antibiotics tested and were therefore considered suitable for further screening as potential starter cultures.

The ability of starter cultures to hydrolyze casein is an important technological feature in cheese production, as proteolysis during ripening influences the release of low-molecular-weight peptides and free amino acids that influence the development of cheese texture and flavor [60]. *Lpb. plantarum* ZI237 and *Lcb. rhamnosus* KO showed the highest degradation of both αs1-casein and β-casein fractions, while β-casein degradation was shown by *Lpb. plantarum* ZI137 and *Lcb. paracasei* KR. Proteolysis of β-casein can generate low-molecular-weight peptides associated with bitterness in cheese, which means that degradation of this protein should be considered with caution [61]. Since the sensory outcome depends on whether such peptides accumulate or are further hydrolyzed, residual bitterness should be further assessed by sensory analysis [62]. Lactobacilli generally exhibit slower milk acidification than other LABs, especially lactococci, mainly due to their less efficient lactose metabolism, which is consistent with the limited acidification activity observed in the strains tested in this study after 6 and 24 h of incubation [63]. From a technological perspective, such a feature does not necessarily represent a disadvantage, especially when the strains are intended for supplemental or probiotic applications, where excessive acidification may negatively affect the technological and sensory properties of the final product [64].

Considering the guidelines stating that a potential probiotic strain must survive the gastrointestinal environment (WHO-FAO 2006), the remaining strains were tested for survival under simulated GIT conditions [65]. The survival rates reported in this study were calculated after each simulated GIT step as a percentage of the initial log CFU/mL value and were used to compare how well each strain maintained its viable cell count relative to the corresponding initial level during sequential GIT exposure. As there is currently no standardized approach to assessing and reporting probiotic survival under gastrointestinal stress, the values obtained should be interpreted in accordance with the analytical and calculation methods used, especially when comparing results between studies [66]. It is important to note that skim milk was chosen as the cell carrier because the literature shows that “free cells” are significantly more sensitive than cells combined with a food carrier [33,67]. The high survival of *Lpb. plantarum* ZI137 and *Lpb. plantarum* ZI237 in gastric juice is in line with previous reports indicating that these species may exhibit particularly high tolerance to these conditions [68,69,70]. This feature is likely due to proven acid tolerance strategies, such as improved proton extrusion and pH homeostasis, accompanied by adaptive changes in membrane composition at low pH values [71,72]. After exposure to duodenal and intestinal juices, the three strains showed high survival rates (41.83% ± 0.75–55.92% ± 5.43), while the significant decrease in viable cells in *Lpb. plantarum* ZI137 can be explained by the composition of these juices that destabilized the bacterial cell membrane by dissociation of the lipid bilayer, leading to leakage of intracellular contents and ultimately cell death [56].

Based on the intact zones of inhibition around the pronase E crystals, it was concluded that the antimicrobial activity of the tested strains was not driven by bacteriocins. Since neutralized cell-free supernatants adjusted to pH 6.5 were not tested, the contribution of organic acids to the observed inhibition could not be distinguished from that of other antimicrobial metabolites commonly produced by lactobacilli [73]. The inhibitory activity of lactobacilli against various pathogens associated with the accumulation of lactic acid and the production of hydrogen peroxide has been reported in previous works [74,75]. It is important to highlight the inhibition of ATCC and laboratory collection strains of *L. monocytogenes* (four out of four strains inhibited the growth of *L. monocytogenes* ATCC 19111 and three out of four inhibited the growth of *L. monocytogenes* IMS4), given that this pathogen is highly adaptable to food-related stress conditions, including low temperatures, NaCl concentrations and modified atmospheres [76]. Inhibition of this pathogen represents a promising biopreservation strategy, and previous studies have already highlighted organic acids and H_2_O_2_ as important factors involved in the control of *L. monocytogenes* [77,78]. Therefore, the antimicrobial profiles observed in this study further support the potential of the selected strains for future applications in food biopreservation.

Adhesion to intestinal epithelial cells is considered an important in vitro criterion in the selection of probiotic candidates [79]. Strains with adhesion ability show beneficial effects in host interactions: they facilitate bacterial–epithelial signaling and potentially limit pathogen binding through competitive exclusion [80]. In this study, all tested lactobacilli adhered efficiently to differentiated Caco-2 cell lines. The values obtained are comparable to those published for previously characterized probiotic candidates [81]. Cellular toxicity represents an important safety consideration in the selection of probiotic candidates since damage to intestinal epithelial cells may reduce cell viability and compromise epithelial barrier integrity, potentially increasing intestinal permeability [82]. In the present study, none of the tested strains significantly increased LDH release, indicating that they did not induce detectable membrane damage under the applied experimental conditions.

*Lpb. plantarum* ZI237 showed the broadest transcriptional effect, with significantly increased mRNA levels in all stages of the autophagy process: initiation (*ULK1*), autophagosome formation (*BECN1*, *PIK3C3*, *AMBRA1*) and autophagosome elongation (*ATG5*, *GABARAP*), as well as significantly decreased mRNA levels of *p62*, which encodes an autophagy receptor and substrate. *Lm. fermentum* KT produced a narrower response, mainly reflected in an increase in *BECN1*, *PIK3C3* and *AMBRA1*, along with a significant decrease in *p62* expression. The most consistent finding was a significant decrease in p62 protein in cells treated with both strains using Western blot analysis, representing the clearest finding of autophagy-related modulation at the protein level. In contrast, Beclin-1 and LC3 showed changes without statistical significance. This partial discrepancy between gene and protein expression is not unexpected since autophagy is regulated at multiple levels and transcript abundance does not necessarily translate into measurable protein accumulation at a single experimental time point [83]. Also, reduced p62 levels alone are not sufficient to conclusively demonstrate increased autophagic flux, especially since the reduction in p62 protein is accompanied by reduced *SQSTM1/p62* mRNA expression. The absence of lysosomal inhibition measurements or other dedicated flux assays precludes the claim that there is a link between increased autophagosome formation and increased lysosomal degradation. Therefore, the present findings support modulation of autophagy-related pathways, but do not provide direct evidence of increased autophagic flux [84].

*Lm. fermentum* KT significantly upregulated the expression of *MUC2*, *MUC5* and *HBD-1*, consistent with previous reports of probiotic-induced modulation of these genes [85,86]. However, these transcriptional changes alone do not demonstrate increased mucus production or enhanced epithelial antimicrobial defense, as the corresponding functional effects were not assessed in the present study.

*Lpb. plantarum* ZI237 and *Lm. fermentum* KT significantly upregulated *NQO1*, which encodes a cytoprotective enzyme involved in redox homeostasis [87,88,89]. Although probiotic-associated antioxidant responses are often discussed in the context of broader Nrf2-related signaling, the observed transcriptional changes suggest that these strains may modulate redox-related pathways through *NQO1* [23]. However, increased *NQO1* expression alone does not provide direct evidence of an antioxidant effect, as functional markers of oxidative stress were not assessed in the present study.

No significant changes in E-cadherin or Claudin-4 protein abundance were detected, while Occludin was significantly reduced in cells treated with *Lm. fermentum* KT (*p* < 0.05). This selective reduction prevents a definitive conclusion that the strain has an overall beneficial effect on epithelial barrier integrity. Additional functional assays, such as transepithelial electrical resistance measurements, are needed to determine the overall effect of *Lm. fermentum* KT on epithelial barrier function.

## 5. Conclusions

This study confirms that traditional Serbian cheeses represent an important reservoir of diverse autochthonous lactic acid bacteria. Seven isolated lactobacilli showed varying degrees of safety, as well as technological and probiotic potential, based on the results. *Lpb. plantarum* ZI237 emerged as the most promising strain due to its safety profile, survival in simulated gastrointestinal conditions, antagonistic activity, proteolytic potential and modulation of genes related to antioxidant defense and autophagy, which was confirmed at the protein level by reduced expression of p62. *Lm. fermentum* KT also emerged as a relevant candidate, especially due to its ability to stimulate genes related to the mucosal barrier, innate epithelial defense and antioxidant defense, while simultaneously showing effects related to autophagy. However, the significant reduction in Occludin protein abundance indicates that the overall effect of *Lm. fermentum* KT on epithelial barrier integrity should be interpreted with caution and requires further investigation using functional barrier assays. Overall, *Lpb. plantarum* ZI237 emerged as the most complete candidate, whereas *Lm. fermentum* KT showed a mixed epithelial response that warrants further evaluation. Nevertheless, additional studies are needed to confirm their performance in food systems and further validate their effects in more complex in vitro and in vivo models.

## Figures and Tables

**Table 1:** List of primers used in this study.

Gene Name	Primer Sequence 5′-3′	Reference
*ULK1_F*	TTTTGTTTCTCCGTTGGGGC	[19]
*ULK1_R*	ACTCTTCCCGGGCTGCTAAT	[19]
*BECN1_F*	CTGGGACAACAAGTTTGACCAT	[35]
*BECN1_R*	GCTCCTCAGAGTTAAACTGGGTT	[35]
*PIK3C3_F*	GCTGTCCTGGAAGACCCAAT	[19]
*PIK3C3_R*	TTCTCACTGGCAAGGCCAAA	[19]
*AMBRA1_F*	GGTGGGAGGAGAGGGGATAG	[19]
*AMBRA1_R*	CGAGGGGCATGTCATCATTT	[19]
*ATG5_F*	CACAAGCAACTCTGGATGGGATTG	[36]
*ATG5_R*	GCAGCCACGGACGAAACAG	[36]
*MAP1LC3B_F*	TTCAGGTTCACAAAACCCGC	[19]
*MAP1LC3B_R*	TCTCACACAGCCCGTTTACC	[19]
*GABARAP_F*	CCCTCGTCCCGCTGATTTTA	[19]
*GABARAP_R*	ATCCCTCCAGCTTGTACCCA	[19]
*GABARAP_F*	CCCTCGTCCCGCTGATTTTA	[19]
*GABARAP_R*	ATCCCTCCAGCTTGTACCCA	[19]
*SQSTM1_F*	GCCAGAGGAACAGATGGAGT	[37]
*SQSTM1_R*	TCCGATTCTG GCATCTGTAG	[37]
*MUC2_F*	CACCTGTGCCCTGGAAGGC	[38]
*MUC2_R*	CGGTCACGTGGGGCAGGTTC	[38]
*MUC5_F*	CGGGTCCACGAGGAGACGGT	[38]
*MUC5_R*	GCTTCTGCAGCCAGGCACGA	[38]
*NQO-1_F*	GGATTGGACCGAGCTGGAAA	[39]
*NQO-1_R*	CAAACTGAAACACCCAGCCG	[39]
*HBD-1_F*	TGTCTGAGATGGCCTCAGGT	[19]
*HBD-1_R*	GGGCAGGCAGAATAGAGACA	[19]
*GAPDH_F*	GTGAAGGTCGGAGTCAACG	[40]
*GAPDH_R*	TGAGGTCAATGAAGGGGTC	[40]

**Table 2:** Identification of lactic acid bacteria isolated from traditional Serbian cheeses based on 16S rRNA gene sequencing.

Sampling Location	Milk Type	Isolate	Species	Accession Number
Čačak	Cow milk	ST137	*Enterococcus lactis*	PZ282880
		ST237	*Lactococcus lactis*	PZ283162
		ST337	*Enterococcus faecium*	PZ306319
		ST242	*Enterococcus durans*	PZ288086
Trstenik	Cow milk	BK537	*Enterococcus durans*	PZ288121
		BK642	*Enterococcus faecium*	PZ288128
Valjevo	Cow milk	TA137	*Leuconostoc mesenteroides*	PZ288129
		TA337	*Leuconostoc mesenteroides*	PZ306430
		TA537	*Leuconostoc mesenteroides*	PZ288160
		TA242	*Enterococcus faecium*	PZ288949
Paraćin	Goat milk	ZI137	*Lactiplantibacillus plantarum*	PZ288173
		ZI237	*Lactiplantibacillus plantarum*	PZ288176
		ZI337	*Lactiplantibacillus plantarum*	PZ288940
		ZI437	*Enterococcus faecium*	PZ306323
		ZI142	*Enterococcus faecium*	PZ288967
Sremska Mitrovica	Goat milk	KZ137	*Lactococcus lactis*	PZ292934
		KZ337	*Lactococcus lactis*	PZ292963
		KZ437	*Enterococcus faecium*	PZ292964
		KZ242	*Leuconostoc mesenteroides*	PZ292966
		KZ342	*Leuconostoc mesenteroides*	PZ292971
Vrbica	Cow milk	AB3	*Lactococcus lactis*	PZ292977
		AB19	*Lactococcus lactis* ssp. *cremoris*	PZ292980
Lajkovac	Cow milk	CD9	*Enterococcus durans*	PZ293150
		CD11	*Enterococcus faecalis*	PZ293156
		CD16	*Streptococcus macedonicus*	PZ295725
Zlatibor	Cow milk	ZL237	*Enterococcus faecium*	PZ295743
Vizić	Cow milk	SAC137	*Enterococcus faecium*	PZ295778
		SAC337	*Enterococcus mundtii*	PZ299402
		SAC437	*Enterococcus faecalis*	PZ299415
		SAC637	*Enterococcus faecalis*	PZ299437
Kopaonik	Cow milk	KO237	*Enterococcus faecalis*	PZ299441
		KO342	*Enterococcus faecium*	PZ299442
		KO542	*Enterococcus faecium*	PZ299445
Pirot	Cow milk	KB	*Lacticaseibacillus paracasei*	PZ299453
		KO	*Lacticaseibacillus rhamnosus*	PZ299454
		KR	*Lacticaseibacillus paracasei*	PZ299458
		KT	*Limosilactibacillus fermentum*	PZ299475

**Table 3:** Minimal inhibitory concentrations (MICs, mg/L) of eight antibiotics for five tested *Lactobacillus* strains. Values outside brackets represent the experimentally determined MICs, whereas values in brackets indicate the corresponding EFSA (2018) breakpoint values [31].

Antibiotic	*Lpb. plantarum* ZI137	*Lpb. plantarum* ZI237	*Lcb. rhamnosus* KO	*Lcb. paracasei* KR	*Lm. fermentum* KT
Amplicillin	≤1 (2)	≤1 (2)	≤2 (4)	≤2 (4)	≤1 (2)
Gentamycin	≤8 (16)	≤8 (16)	≤8 (16)	≤16 (32)	≤8 (16)
Kanamycin	≤32 (64)	≤32 (64)	≤32 (64)	≤32 (64)	≤32 (64)
Streptomycin	n.r.	n.r.	≤16 (32)	≤32 (64)	≤32 (64)
Erythromycin	≤0.5 (1)	≤0.5 (1)	≤0.5 (1)	≤0.5 (1)	≤0.5 (1)
Clindamycin	≤2 (4)	≤2 (4)	≤2 (4)	≤2 (4)	≤2 (4)
Tetracycline	≤16 (32)	≤16 (32)	≤4 (8)	≤2 (4)	≤4 (8)
Chloramphenicol	≤4 (8)	≤4 (8)	≤2 (4)	≤2 (4)	≤2 (4)

Note: n.r., not required.

**Table 4:** Antimicrobial activity against indicator pathogenic microorganisms.

	*Lpb. plantarum* ZI137	*Lpb. plantarum* ZI237	*Lcb. rhamnosus* KO	*Lm. fermentum* KT
*Salmonella enterica* subsp. *enterica* serovar Typhimurium ATCC 14028	+	+	+	+
*Streptococcus pyogenes* ATCC 19615	+	−	+	−
*Staphylococcus aureus* ATCC 25923	−	+	−	+
*Escherichia coli* ATCC 25922	+	+	+	−
*Listeria monocytogenes* ATCC 19111	+	+	+	−
*Listeria monocytogenes* IMS4	+	+	+	−

Note: + positive reaction; − no activity.

## Data Availability

The sequence data generated in this study have been deposited in GenBank under accession numbers ranging from PZ282880 to PZ306430. Other datasets generated and/or analyzed during the current study are available from the corresponding author upon reasonable request.

## Supplementary Materials

The following supporting information can be downloaded at: https://www.mdpi.com/article/10.3390/foods15183220/s1, Figure S1: Images of LC3-I/LC3-II detection. Corresponding Beta-Actin loading controls are shown, with membrane edges visible to indicate the full extent of each blot. The membrane was physically cut into strips prior to primary antibody incubation. Protein bands selected for densitometric analysis were identified and marked according to their expected molecular mass (kDa) based on the protein ladder; Figure S2: Images of Beclin detection. Corresponding Beta-Actin loading controls are shown, with membrane edges visible to indicate the full extent of each blot. The membrane was physically cut into strips prior to primary antibody incubation. Protein bands selected for densitometric analysis were identified and marked according to their expected molecular mass (kDa) based on the protein ladder; Figure S3: Images of p62 detection. Corresponding Beta-Actin loading controls are shown, with membrane edges visible to indicate the full extent of each blot. The membrane was physically cut into strips prior to primary antibody incubation. Protein bands selected for densitometric analysis were identified and marked according to their expected molecular mass (kDa) based on the protein ladder; Figure S4: Images of Claudin detection. Corresponding Beta-Actin loading controls are shown, with membrane edges visible to indicate the full extent of each blot. The membrane was physically cut into strips prior to primary antibody incubation. Protein bands selected for densitometric analysis were identified and marked according to their expected molecular mass (kDa) based on the protein ladder; Figure S5: Images of Occludin detection. Corresponding Beta-Actin loading controls are shown, with membrane edges visible to indicate the full extent of each blot. The membrane was physically cut into strips prior to primary antibody incubation. Protein bands selected for densitometric analysis were identified and marked according to their expected molecular mass (kDa) based on the protein ladder; Figure S6: Images of E-cadherin detection. Corresponding Beta-Actin loading controls are shown, with membrane edges visible to indicate the full extent of each blot. The membrane was physically cut into strips prior to primary antibody incubation. Protein bands selected for densitometric analysis were identified and marked according to their expected molecular mass (kDa) based on the protein ladder.
