# Supplementary material for: Biodiversity of NSLAB in Traditional Serbian Cheeses and Stepwise Functional Characterization of Selected Autochthonous Lactobacillus Strains Through Technological, Probiotic and Intestinal Epithelial Interaction Assessment
Source: Foods. 2026 Sep 11;15(18):3220. doi: 10.3390/foods15183220 (PMC13606762; doi:10.3390/foods15183220)
Supplement: Supplementary file 1 [file foods-15-03220-s001.zip › foods-4504680-supplementary.pdf]

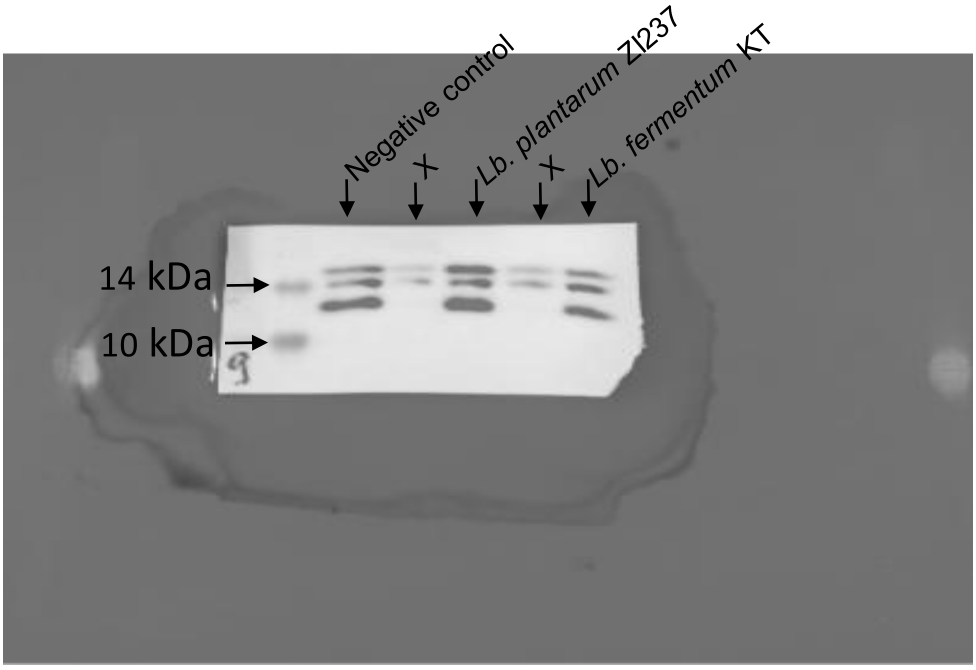

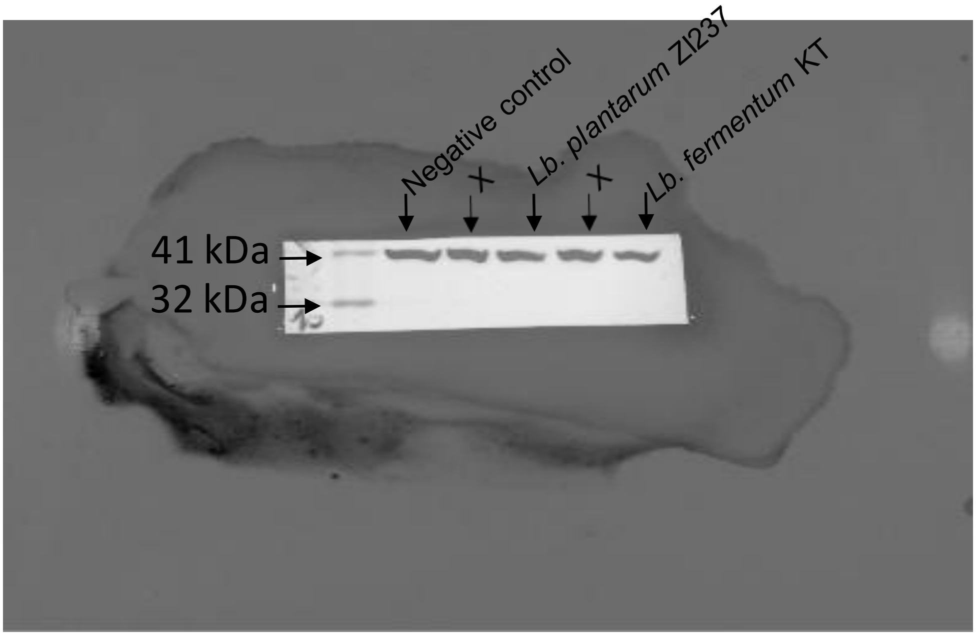

Figure S1. Images of LC3-I/LC3-II detection. Corresponding Beta-Actin loading controls are shown, with membrane edges visible to indicate the full extent of each blot.
The membrane was physically cut into strips prior to primary antibody incubation.
Protein bands selected for densitometric analysis were identified and marked according to their expected molecular mass (kDa) based on the protein ladder.

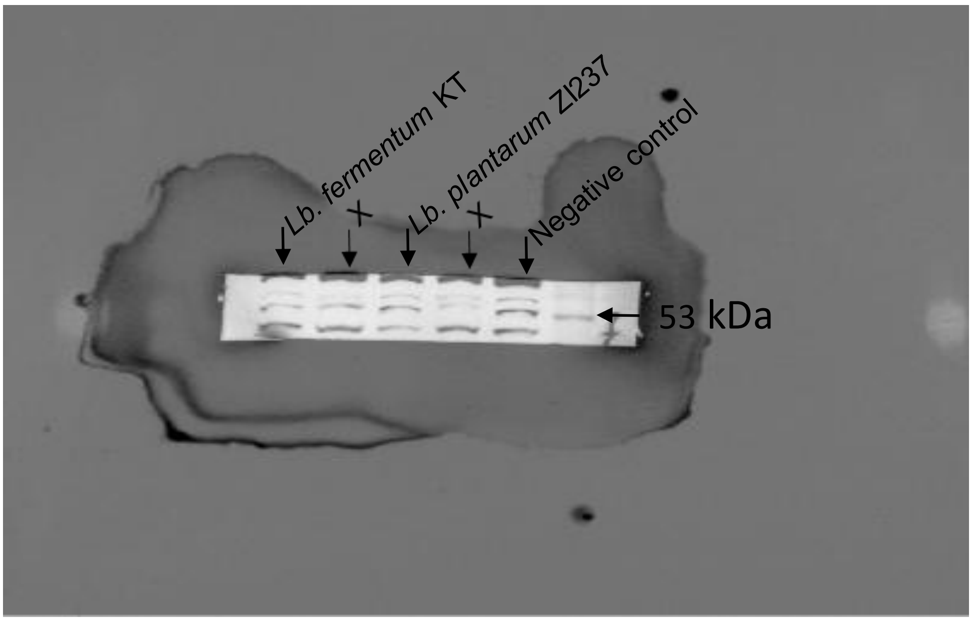

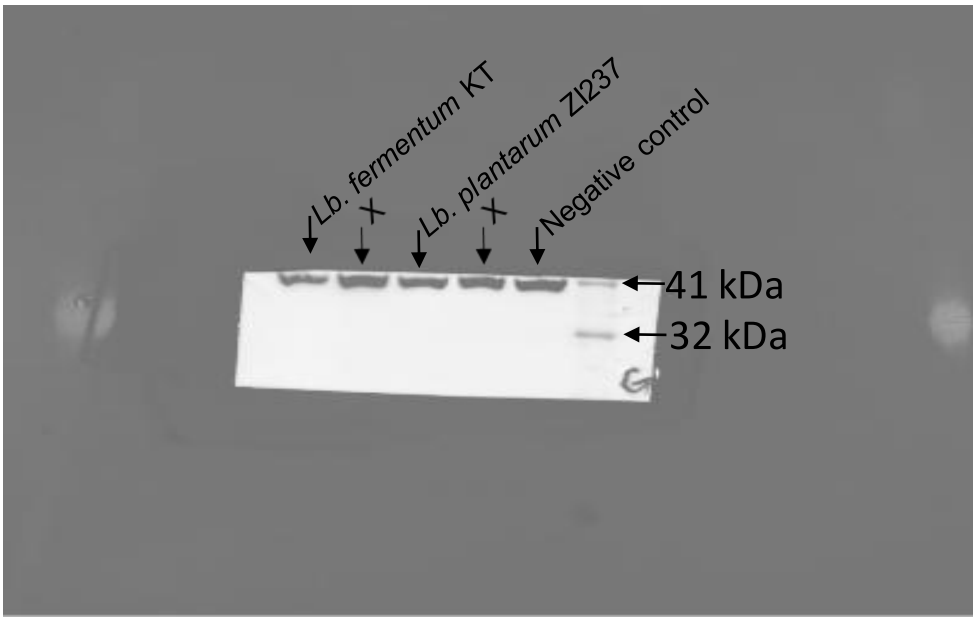

Figure S2. Images of Beclin detection. Corresponding Beta-Actin loading controls are shown, with membrane edges visible to indicate the full extent of each blot.

The membrane was physically cut into strips prior to primary antibody incubation.

Protein bands selected for densitometric analysis were identified and marked according to their expected molecular mass (kDa) based on the protein ladder.

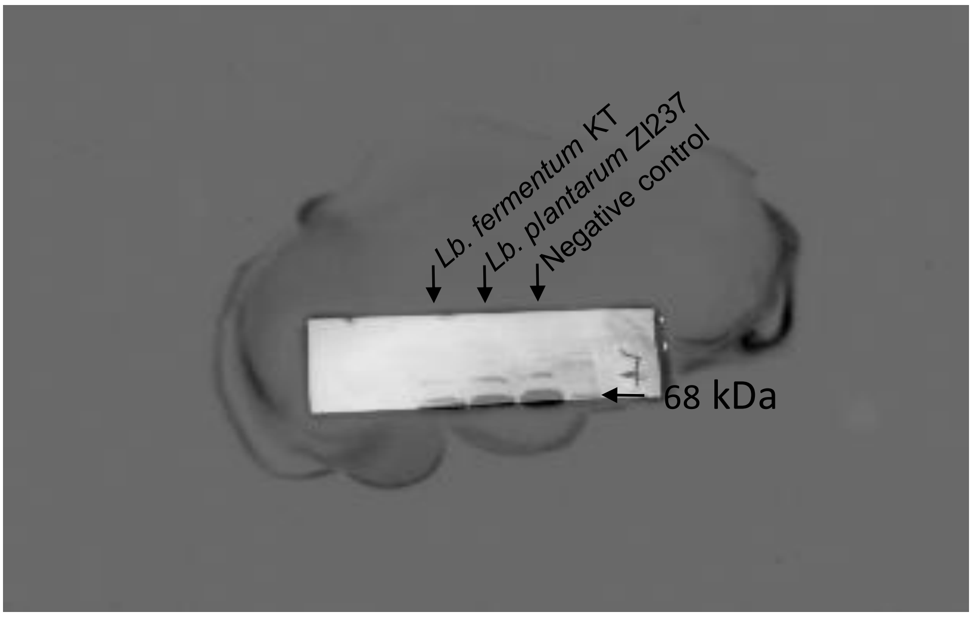

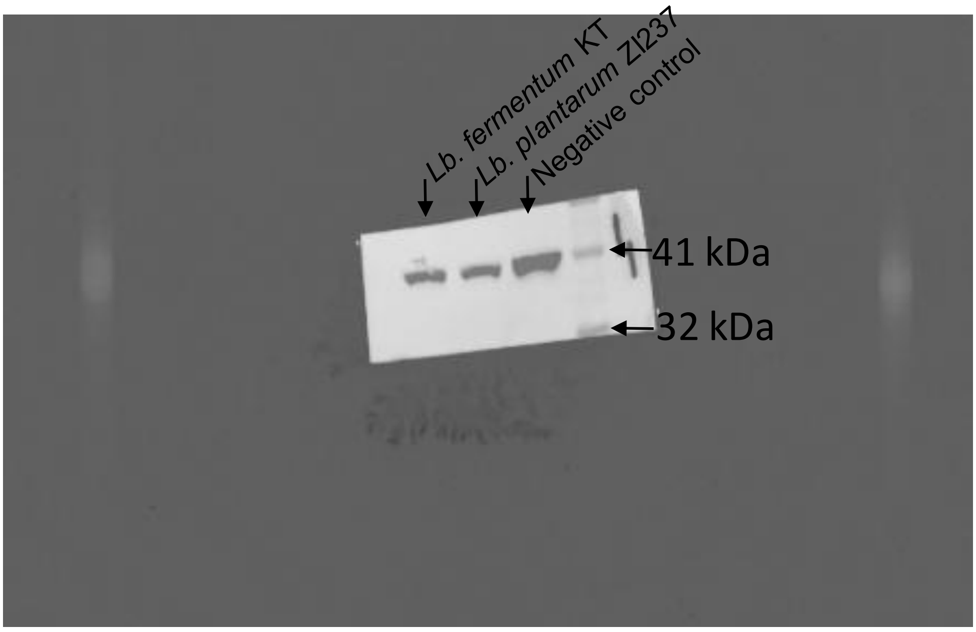

Figure S3. Images of p62 detection. Corresponding Beta-Actin loading controls are shown, with membrane edges visible to indicate the full extent of each blot.

The membrane was physically cut into strips prior to primary antibody incubation.

Protein bands selected for densitometric analysis were identified and marked according to their expected molecular mass (kDa) based on the protein ladder.

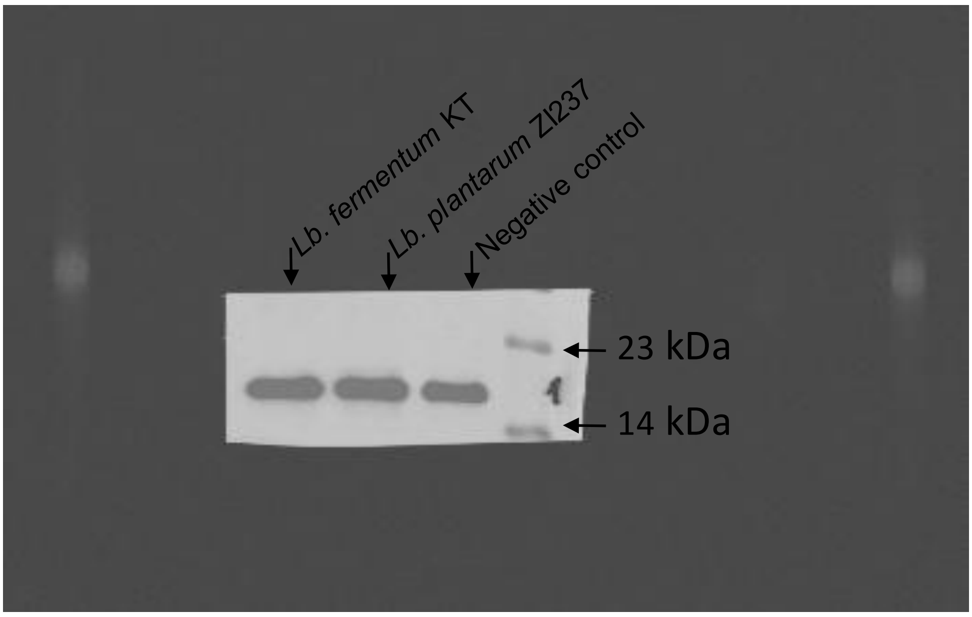

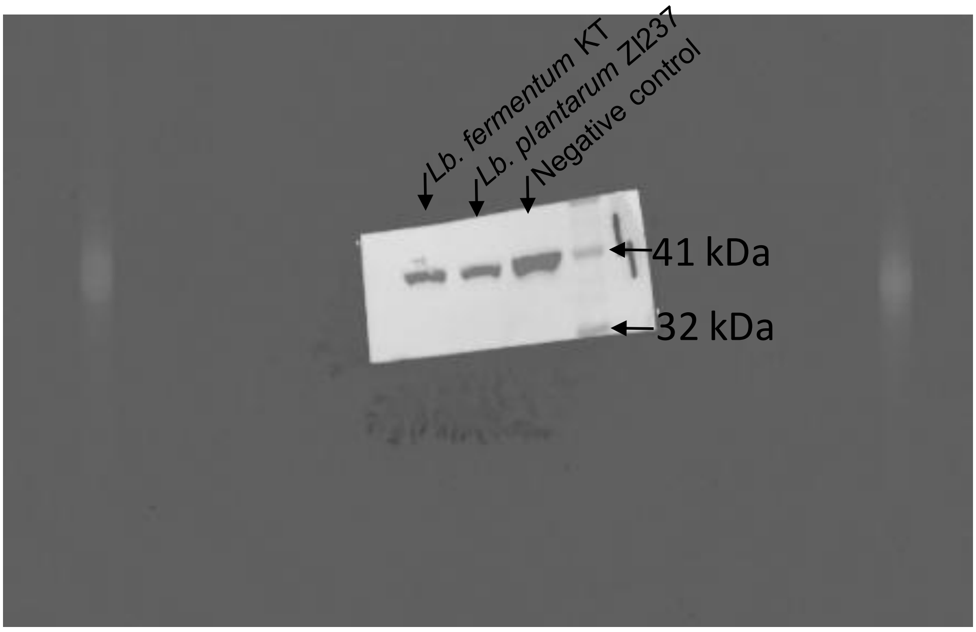

Figure S4. Images of Claudin detection. Corresponding Beta-Actin loading controls are shown, with membrane edges visible to indicate the full extent of each blot.

The membrane was physically cut into strips prior to primary antibody incubation.

Protein bands selected for densitometric analysis were identified and marked according to their expected molecular mass (kDa) based on the protein ladder.

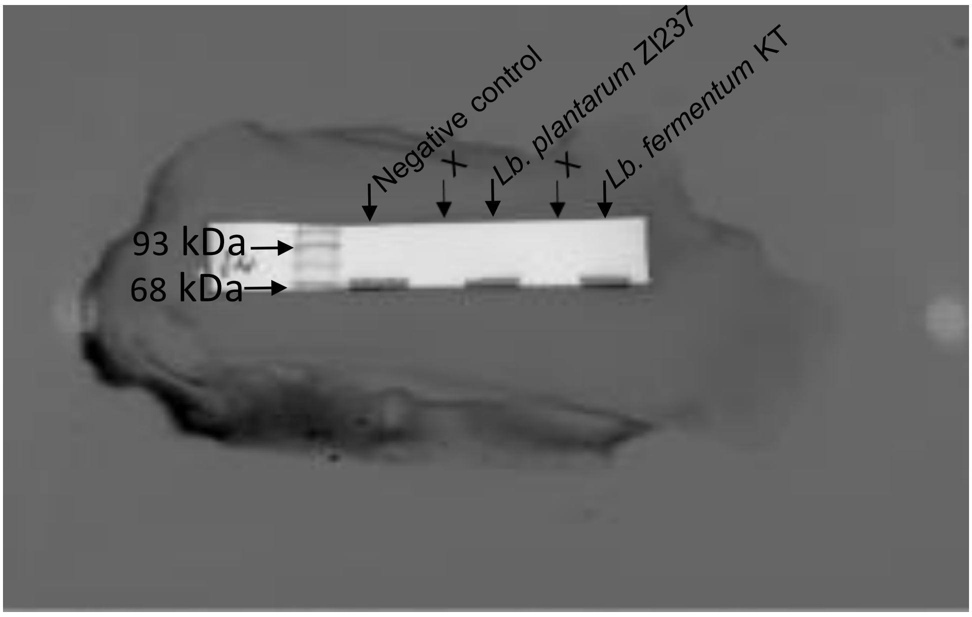

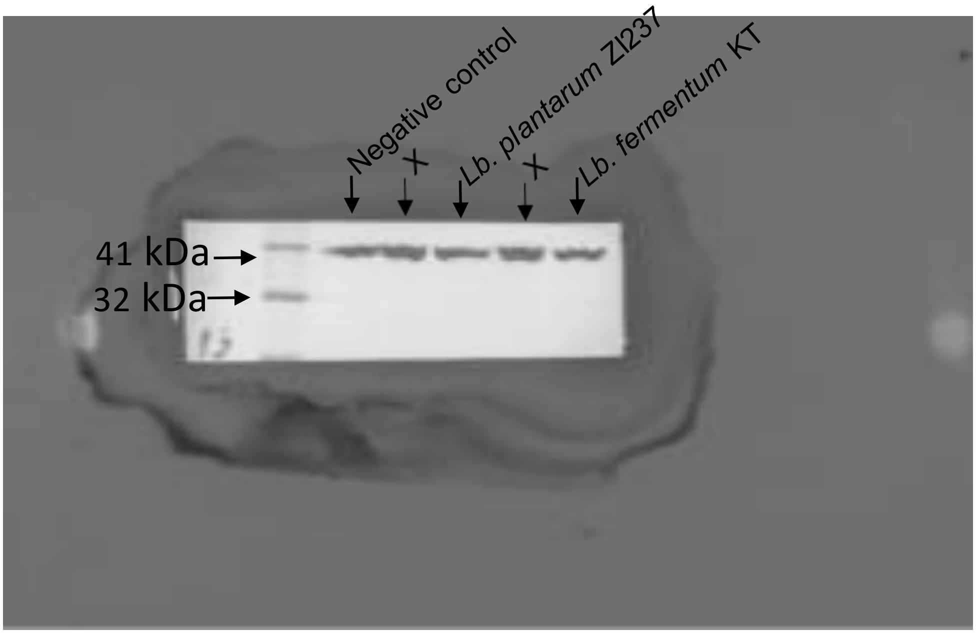

Figure S5. Images of Occludin detection. Corresponding Beta-Actin loading controls are shown, with membrane edges visible to indicate the full extent of each blot.

The membrane was physically cut into strips prior to primary antibody incubation.

Protein bands selected for densitometric analysis were identified and marked according to their expected molecular mass (kDa) based on the protein ladder.

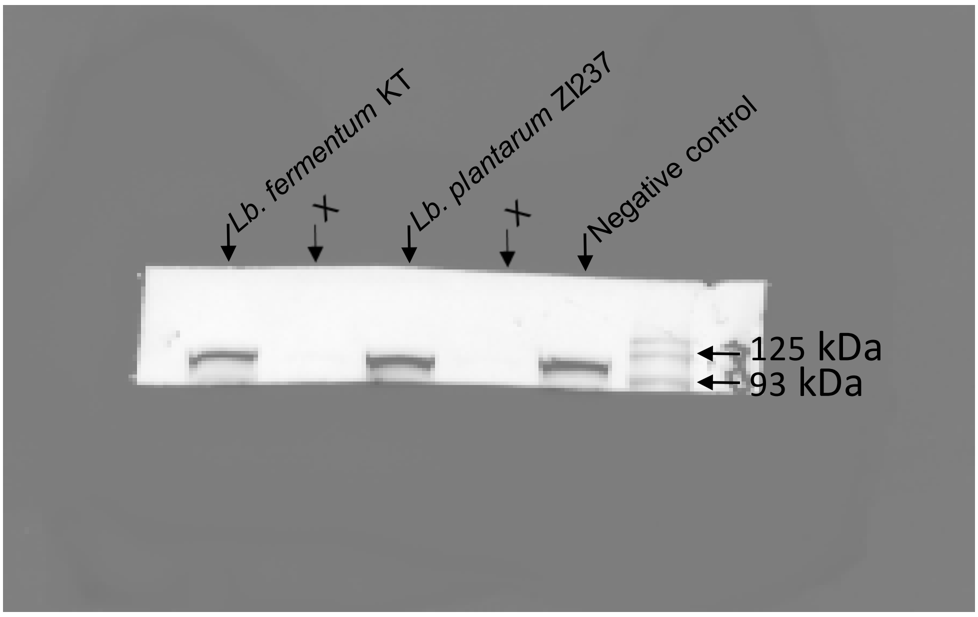

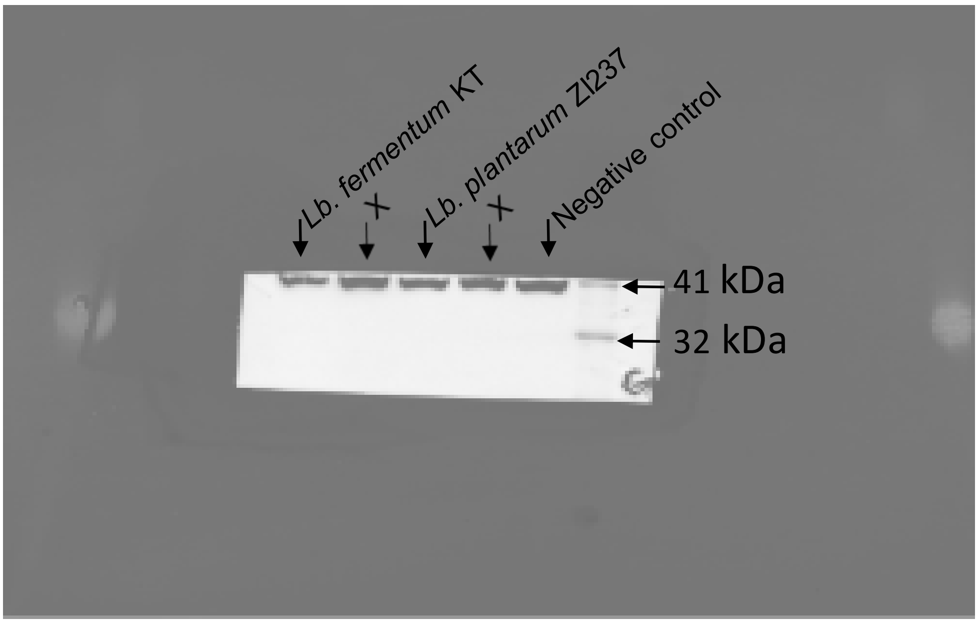

Figure S6. Images of E-cadherin detection. Corresponding Beta-Actin loading controls are shown, with membrane edges visible to indicate the full extent of each blot.

The membrane was physically cut into strips prior to primary antibody incubation.

Protein bands selected for densitometric analysis were identified and marked according to their expected molecular mass (kDa) based on the protein ladder.
